# Incremental value of renal, metabolic, and inflammatory markers for prevalent self-reported cardiovascular disease across NHANES cycles: A repeated cross-sectional validation study

**DOI:** 10.1097/MD.0000000000051425

**Published:** 2026-11-06

**Authors:** Haoyang Li, Xiang Li, Xuefeng Shi

**Affiliations:** a Division of Cardiology, Qinghai University Affiliated Hospital, Xining, Qinghai, China; b Department of Thyroid and Breast Surgery, Qinghai University Affiliated Hospital, Xining, Qinghai, China; c Department of Respiratory and Critical Care Medicine, Qinghai Provincial People’s Hospital, Xining, Qinghai, China.

**Keywords:** Albuminuria, Cardiovascular Diseases, Cross-Sectional Studies, National Health and Nutrition Examination Survey, Renal Insufficiency, Validation Study

## Abstract

Renal dysfunction, metabolic stress, and systemic inflammation are associated with cardiovascular disease (CVD), but their incremental value beyond routine clinical factors is uncertain. This repeated cross-sectional study used 7 National Health and Nutrition Examination Survey (NHANES) cycles (2005–2018) for development and leave-one-cycle-out validation. The primary outcome was prevalent self-reported CVD. A locked clinical core was extended with estimated glomerular filtration rate plus log2 urine albumin-to-creatinine ratio (renal), triglyceride-glucose waist circumference index (TyG-WC; metabolic), systemic inflammation response index (inflammatory), or all markers. Elastic Net was primary and XGBoost secondary. Preprocessing, imputation, and full-grid tuning were confined to training cycles. Survey-aware 95% confidence intervals (CIs) used 2000 Rao-Wu bootstrap replicates. NHANES 2021–2023 underwent a separate temporal assessment, corrected after inspection of initial outputs, using development-trained pipelines without temporal tuning or recalibration. Development examination and fasting samples included 37,482 adults (4222 CVD events) and 15,956 adults (1813 events), respectively. The Elastic Net core area under the receiver operating characteristic curve (AUROC) was 0.8490 (95% CI, 0.8410–0.8560). Renal markers improved AUROC by 0.0046 (0.0031–0.0062) and area under the precision-recall curve (PR-AUC) by 0.0161 (0.0101–0.0223); the combined extension improved AUROC by 0.0072 (0.0039–0.0106). In the corrected 2021–2023 assessment, phlebotomy and fasting samples included 5755 adults (739 events) and 2940 adults (355 events). Core AUROC was 0.8533 (0.8313–0.8753). Renal incremental AUROC was 0.0041 (0.0001–0.0081) and PR-AUC was 0.0217 (0.0093–0.0310); combined incremental AUROC was 0.0074 (0.0016–0.0124). TyG-WC alone showed no clear increment. XGBoost gains were less consistent across cycles and temporal metrics. Renal and combined extensions showed modest, algorithm-dependent gains in classifying prevalent self-reported CVD. The corrected temporal assessment provided supportive evidence for Elastic Net. These findings describe cross-sectional association patterns; prospective studies with adjudicated outcomes and defined clinical decisions are needed to evaluate clinical utility.

## 1. Introduction

Cardiovascular disease (CVD) remains a major source of morbidity and mortality, with disease burden distributed across demographic, behavioral, metabolic, vascular, and renal domains. Established factors such as age, smoking, blood pressure, diabetes, adiposity, and circulating lipids provide a strong baseline for identifying people with CVD. The practical question is how much information additional biomarkers contribute beyond this clinical core and how consistently that increment recurs across survey periods.

Kidney markers are plausible candidates for such incremental information. Estimated glomerular filtration rate (eGFR) and albuminuria describe related but distinct aspects of renal and vascular health, and both have been associated with cardiovascular outcomes across general-population cohorts and can add discrimination beyond traditional factors.^[1–4]^ The triglyceride-glucose (TyG) index was developed as a surrogate of insulin resistance,^[5]^ and its combination with waist circumference has been associated cross-sectionally with CVD in NHANES.^[6]^ The systemic inflammation response index (SIRI), calculated from neutrophil, monocyte, and lymphocyte counts, has likewise been associated with coronary heart disease in NHANES.^[7]^ These markers are routinely obtainable, but biological overlap and correlated predictors can make apparent gains sample-dependent.

Many published biomarker analyses emphasize association, feature importance, or performance within a single pooled sample. Incremental evaluation additionally requires a fixed comparator, matched participants and weights, and assessment across survey periods. Performance estimates may be optimistic when imputation, preprocessing, or tuning uses information from the evaluation period. Repeatedly holding out an entire NHANES cycle tests period-to-period reproducibility while retaining the national survey structure.^[8,9]^

We therefore evaluated the incremental value of renal, metabolic, and inflammatory marker domains for identifying prevalent self-reported CVD using prespecified leave-one-cycle-out validation in NHANES 2005–2018, followed by a separate postaudit corrected temporal assessment in NHANES 2021–2023. We prespecified a core clinical model and 4 extensions, used Elastic Net logistic regression as the primary algorithm and XGBoost as a nonlinear sensitivity analysis,^[10,11]^ and confined all data-dependent processing to development cycles. We assessed discrimination, probabilistic accuracy, calibration, survey-aware uncertainty, across-cycle random-effects summaries, missing-UACR and class-imbalance sensitivities, exploratory decision curves, and temporal application of the frozen pipelines to the later survey period.

## 2. Methods

### 2.1. Study design and population

This repeated cross-sectional study used public-use NHANES data from seven two-year cycles spanning 2005–2006 to 2017–2018. NHANES uses a complex, multistage probability design; survey methods and analytic guidance are provided by the US National Center for Health Statistics (NCHS).^[12]^ Eligible participants were aged 20 years or older, were not pregnant where pregnancy status was available, had an ascertainable CVD outcome, and had valid domain-specific survey design variables. The mobile examination center (MEC)-based core analysis contained 37,482 participants, whereas models requiring fasting triglycerides and glucose used the fasting subsample of 15,956 participants. NHANES 2021–2023 used a new sample design after pandemic-related fieldwork disruption and was therefore treated as a separate temporal period rather than pooled with earlier cycles.^[13]^ All NHANES protocols were approved by the National Center for Health Statistics Research Ethics Review Board, and all participants provided written informed consent.^[14]^ This secondary analysis used de-identified public-use data and involved no additional participant contact.

Before final model fitting, we prespecified the variables, transformations, survey weights, missing-data rules, validation folds, hyperparameter grids, and sensitivity analyses. Development analyses used only 2005–2018; NHANES 2021–2023 remained unavailable for analysis until development validation and sensitivity analyses were complete. Reporting drew on the Transparent Reporting of a Multivariable Prediction Model for Individual Prognosis Or Diagnosis plus Artificial Intelligence (TRIPOD + AI) statement.^[15]^ We also followed the Strengthening the Reporting of Observational Studies in Epidemiology (STROBE) statement, adapting terminology to cross-sectional classification.^[16]^

### 2.2. Study size, fairness, and patient involvement

The study included all eligible public-use NHANES participants in the prespecified cycles. Sociodemographic variables were retained in the core model, and distributions by outcome and survey period are reported descriptively. Fairness adjustments, protected-group model updating, and subgroup optimization were outside the prespecified analysis. No patients or members of the public were involved in the design, conduct, interpretation, or dissemination planning of this secondary analysis.

### 2.3. Outcome

The outcome was prevalent self-reported CVD. Participants were classified as cases when any observed component indicated that a health professional had diagnosed congestive heart failure, coronary heart disease, angina, heart attack or myocardial infarction, or stroke. Participants were classified as noncases when no observed component was positive and at least 1 component was observed; records with all 5 components missing were excluded. Because self-report agreement differs by cardiovascular condition,^[17]^ a strict sensitivity retained cases with any positive component but required all 5 components to be observed and negative for classification as a noncase. Outcome components were never included as predictors.

The outcome captured disease history at examination through the standardized NHANES questionnaire, without study-specific adjudication or a prospective prediction horizon.

### 2.4. Predictor sets and derived markers

Model 0 was the locked core clinical model and included age, sex, race/ethnicity, education, smoking status, body mass index, mean systolic blood pressure, diabetes status, total cholesterol, and high-density lipoprotein cholesterol. Diabetes was defined by self-report, glycated hemoglobin of at least 6.5%, or fasting glucose of at least 126 mg/dL. Smoking was categorized as never, former, or current. Mean systolic blood pressure was calculated from available valid examination readings.

Model 1 added eGFR and log2-transformed urine albumin-to-creatinine ratio (UACR). eGFR used the 2021 Chronic Kidney Disease Epidemiology Collaboration (CKD-EPI) race-free creatinine equation.^[4]^ UACR was taken from the NHANES direct value when available or calculated from urine albumin and creatinine, and the base-2 logarithm represented the contrast per doubling. Model 2 added TyG-WC, calculated as ln[fasting triglycerides (mg/dL) × fasting glucose (mg/dL)/ 2] multiplied by waist circumference in centimeters.^[5,6]^ Model 3 added SIRI, calculated as absolute neutrophil count × absolute monocyte count/ absolute lymphocyte count.^[7]^ Model 4 added all renal, metabolic, and inflammatory components. C-reactive protein, outcome components, and outcome-selected features were excluded. The cycle-specific variable definitions and harmonization are documented in Table S9, Supplemental Digital Content 1.

### 2.5. Survey domains and weights

For 2005 to 2018, mobile examination center weights were used for Models 0, 1, and 3, and fasting-subsample weights for Models 2 and 4. For 2021 to 2023, phlebotomy weights (WTPH2YR) were used for Models 0, 1, and 3, and fasting-subsample weights (WTSAF2YR) for Models 2 and 4, following cycle-specific NCHS guidance.^[13]^ SAS special-missing weight values represented as tiny positive numbers were treated as missing by requiring analysis weights >1e-8. Seven-cycle development weights divided each 2-year weight by 7. Fitting weights were normalized to a mean of 1 within each training fold. Every paired incremental comparison used identical participants, the extended model’s domain weight, and shared survey-replicate factors. Design-based uncertainty accounted for cycle, masked variance stratum, and primary sampling unit.^[18]^

### 2.6. Missing data and preprocessing

All preprocessing was fitted within the training data for each validation split. Continuous predictors were median-imputed; categorical predictors received an explicit Missing level and were encoded with OneHotEncoder(drop = None, handle_unknown=“ignore”), retaining all observed levels. Continuous variables were standardized for Elastic Net using training-data means and standard deviations; XGBoost used unscaled continuous inputs. Categorical coefficients in Table S11, Supplemental Digital Content 2 are therefore penalized joint-model terms, not conventional unadjusted contrasts against an omitted reference category. No outcome information from a held-out cycle informed imputation, scaling, transformation, or model selection.

### 2.7. Algorithms and hyperparameter selection

The primary algorithm was Elastic Net logistic regression with the saga solver.^[10]^ The locked grid crossed five l1 ratios (0, 0.25, 0.50, 0.75, and 1.0) with 9 inverse-regularization values (C = 0.001, 0.003, 0.01, 0.03, 0.1, 0.3, 1, 3, and 10), yielding 45 candidates per task. The secondary algorithm was XGBoost binary logistic classification^[11]^ with a finite grid over tree depth, child weight, learning rate, number of estimators, row and column subsampling, and L1 and L2 regularization. The main analyses used the observed class distribution; no synthetic oversampling was performed. Selected hyperparameters and model hashes are reported in Table S10, Supplemental Digital Content 3.

Outer validation left 1 entire NHANES cycle out in turn. Within the remaining 6 cycles, inner validation again left 1 training cycle out. Hyperparameters were selected by survey-weighted log loss across inner-cycle predictions, with weighted Brier score prespecified as a key secondary metric. The outer cycle was used only once to generate held-out probabilities after the pipeline and hyperparameters had been fixed. This produced 7 mutually exclusive out-of-cycle prediction sets for every model and algorithm. This internal-external validation structure follows recommendations for evaluating transport across clustered settings.^[8,9]^

### 2.8. Performance and incremental value

Performance measures were survey-weighted area under the receiver operating characteristic curve (AUROC), area under the precision-recall curve (PR-AUC), Brier score, log loss, calibration intercept, and calibration slope. PR-AUC emphasizes positive-class retrieval when outcomes are imbalanced.^[19]^ Calibration intercept and slope were interpreted using the established calibration hierarchy.^[20]^ Incremental effects were calculated as extended minus paired core for AUROC and PR-AUC, and as core minus extended for Brier score and log loss. Positive values therefore indicated improvement, with every comparison using matched participants and weights. Survey-bootstrap confidence intervals were conditional on fixed out-of-cycle predictions and captured survey-sampling variability rather than uncertainty from repeated preprocessing, tuning, and model fitting.

Design-aware confidence intervals used 2000 deterministic Rao-Wu rescaled bootstrap replicates. Primary sampling units were resampled within cycle-specific masked strata, and the same replicate factor was shared across paired predictions. Two-sided 95% percentile intervals were reported. Pooled model performance was the equal-cycle mean of cycle-specific estimators; pooled incremental value was the participant-pooled paired estimator across all development cycles.

### 2.9. Sensitivity, meta-analysis, and decision curves

For Models 1 and 4, UACR handling was examined using complete-case refitting, nested multiple imputation, and stabilized inverse-probability weighting, in addition to the main fold-median pipeline. Multiple imputation used 20 posterior draws from a Bayesian-ridge iterative imputer fitted within each outer training fold; the outcome was excluded from imputation. The inverse-probability model was fitted in the training fold without UACR, with prespecified propensity clipping and weight capping. Main hyperparameters were held fixed.

Class-imbalance sensitivity used training-derived inverse-frequency class weights for Elastic Net and the training negative-to-positive ratio for XGBoost. Class counts were preserved without synthetic oversampling or other resampling. Cycle-specific incremental effects were pooled using restricted maximum-likelihood random-effects models with modified Hartung-Knapp confidence intervals. Exploratory decision-curve analysis used pooled 2005–2018 out-of-cycle probabilities and evaluated thresholds from 0.01 to 0.30 and compared core, extended, treat-all, and treat-none strategies, following the decision-curve framework described by Vickers and Elkin.^[21]^ Net-benefit uncertainty used the same paired survey bootstrap replicates. Sensitivity analyses were evaluated separately from the frozen main models. The strict outcome-definition sensitivity evaluated unchanged primary Elastic Net predictions after excluding partial nonpositive response patterns; the models remained fixed. No multiplicity adjustment was applied across model extensions, algorithms, performance metrics, sensitivity analyses, or decision-curve thresholds. The interval estimates provide descriptive and supportive evidence.

### 2.10. Postaudit corrected temporal assessment

After development-period validation and model freezing, Stage 3B applied the development pipelines to NHANES 2021–2023 without temporal tuning, refitting, recalibration, or threshold selection. An implementation audit after the first temporal run identified 2 errors. Metabolic and combined comparisons used a mobile-examination-center-trained core instead of the prespecified fasting-trained core; blood-analyte models used examination rather than phlebotomy weights. The comparator was reconstructed using only 2005 to 2018 data and the unchanged locked grid; component-specific eligibility was corrected, and all temporal estimates were recomputed. Original outputs were archived with hashes. The corrections followed inspection of the initial temporal outputs, so the corrected assessment lacked prospective blinding and provides supportive evidence.

The corrected temporal analysis used the same probability metrics and paired direction conventions as development. Ninety-five percent CIs used 2000 deterministic Rao-Wu rescaled bootstrap replicates within 2021 to 2023 masked strata and primary sampling units. Each comparison shared participants, weights, and replicate factors. Corrections followed NCHS weight guidance and the prespecified same-sample comparator rule, while model selection remained confined to development data.

### 2.11. Software and reproducibility

Data processing, model development, bootstrap inference, meta-analysis, decision curves, tables, and figures were implemented in Python using version-controlled scripts. All prespecified model-selection grids were completed. Final development model objects and the paired fasting-core comparators were verified by SHA-256 hashes, and pilot candidate-limited runs were excluded from manuscript results. The frozen development-model inventory is reported in Table S4, Supplemental Digital Content 4.

Model outputs were individual probabilities of prevalent CVD; decision thresholds were exploratory. The frozen model objects, feature lists, hyperparameters, aggregate result tables, figure source data, and analytical code are available through the permanent GitHub release v1.1.1 , archived in Zenodo at https://doi.org/10.5281/zenodo.22177343; Supplemental Data File S1, Supplemental Digital Content 5 provides repository-access information, a SHA-256 file inventory, and machine-readable source data for all main and supplemental figures.

## 3. Results

### 3.1. Analytic sample and validation framework

Across the 7 development cycles, 39,033 adults had an ascertainable CVD outcome before analysis-weight eligibility. The final examination sample included 37,482 participants and 4222 CVD events, and the fasting sample included 15,956 participants and 1813 events. In 2021 to 2023, the corrected phlebotomy sample included 5755 participants with 739 events, and the fasting sample included 2940 participants with 355 events (Fig. 1, Table S5, Supplemental Digital Content 6). Each development cycle served once as the outer validation set, and all 49 outer tasks per algorithm – seven task types across 7 held-out cycles, including 2 fasting-matched core tasks – completed their full locked grids.

**Figure 1.**
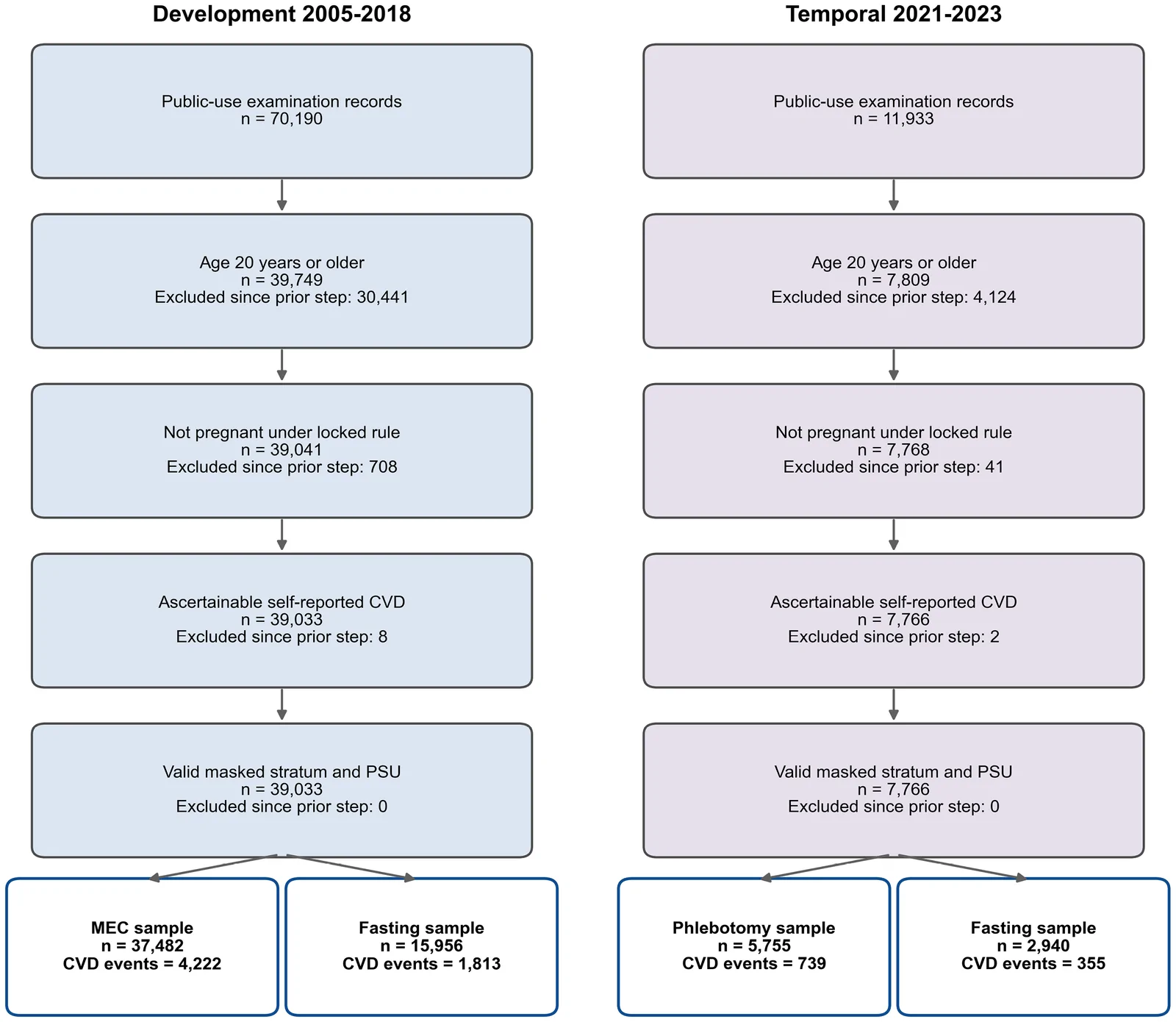
Participant flow by analysis period and survey domain. Counts show locked eligibility steps and the final development examination and fasting domains and corrected 2021–2023 phlebotomy and fasting domains. SAS special-missing weights represented as tiny positive values were excluded with the greater-than-1e-8 rule. Fasting is a domain-specific subsample rather than attrition after model fitting.

Survey-weighted participant characteristics showed the expected separation by prevalent CVD without formal baseline-significance testing (Table 1). In both periods, participants reporting CVD were older, had more diabetes, lower mean eGFR, and higher median UACR than those without CVD. In 2021 to 2023, the CVD and non-CVD groups had mean ages of 65.9 and 47.2 years, diabetes prevalences of 41.1 and 12.4%, mean eGFR values of 77.3 and 97.1 mL/min/1.73 m^2^, and median UACR values of 12.5 and 7.1 mg/g, respectively. Predictor missingness is reported in Table S7, Supplemental Digital Content 7.

**Table 1:** Survey-weighted participant characteristics by cardiovascular disease status and analysis period.

Characteristic	Development 2005–2018: No CVD	Development 2005–2018: CVD	Temporal 2021–2023: No CVD	Temporal 2021–2023: CVD
**Primary component-weight sample**				
Unweighted n	33,260	4222	5016	739
Age, years	45.9 (16.3)	65.0 (13.4)	47.2 (16.9)	65.9 (12.2)
Female, %	51.8	46.1	51.9	44.0
Race/ethnicity: Mexican American, %	8.8	4.6	7.4	3.8
Race/ethnicity: Other Hispanic, %	5.8	3.5	9.8	7.6
Race/ethnicity: Non-Hispanic White, %	66.3	73.1	59.2	68.9
Race/ethnicity: Non-Hispanic Black, %	11.3	12.1	11.1	10.3
Race/ethnicity: Non-Hispanic Asian, %	3.4	1.7	6.4	3.2
Race/ethnicity: Other/Multi, %	4.5	5.0	6.0	6.2
Education: <9th grade, %	5.3	9.8	3.5	6.7
Education: 9–11th grade, %	10.3	14.4	5.6	12.5
Education: High school/GED, %	23.0	27.3	25.7	32.3
Education: Some college/AA, %	31.4	29.1	29.5	29.7
Education: College graduate+, %	30.0	19.4	35.7	18.9
Education missing, %	0.1	0.0	0.0	0.0
Smoking: Never, %	56.5	39.5	64.2	38.5
Smoking: Former, %	23.1	39.0	21.8	40.2
Smoking: Current, %	20.3	21.5	13.9	21.3
Smoking missing, %	0.0	0.0	0.1	0.0
Body mass index, kg/m2	28.9 (6.8)	30.6 (7.2)	29.5 (7.2)	31.5 (7.7)
Systolic blood pressure, mm Hg	121.8 (16.9)	130.0 (20.9)	120.5 (16.4)	127.2 (21.4)
Diabetes, %	10.6	36.5	12.4	41.1
Total cholesterol, mg/dL	195.1 (40.7)	180.1 (45.0)	189.8 (40.9)	170.9 (49.4)
HDL cholesterol, mg/dL	53.7 (16.4)	50.5 (16.3)	53.9 (14.5)	50.9 (13.9)
eGFR, mL/min/1.73 m2	97.2 (19.9)	76.3 (23.4)	97.1 (19.8)	77.3 (22.4)
UACR, mg/g	6.4 (4.3–11.3)	10.8 (5.9–29.1)	7.1 (4.7–12.4)	12.5 (6.8–36.1)
SIRI	1.03 (0.72–1.48)	1.33 (0.90–2.00)	0.98 (0.68–1.46)	1.36 (0.88–2.10)
**Fasting subsample**				
Unweighted fasting n	14,143	1813	2585	355
Waist circumference, cm	98.5 (16.4)	106.1 (16.5)	99.6 (16.8)	107.4 (16.0)
Fasting glucose, mg/dL	105.1 (28.9)	120.6 (42.4)	107.2 (34.4)	123.4 (38.9)
Triglycerides, mg/dL	100.0 (68.0–147.0)	115.0 (81.0–167.0)	80.8 (54.3–121.9)	96.5 (70.0–138.5)
TyG-WC	833.5 (715.4–957.5)	923.4 (806.9–1059.3)	818.6 (705.2–956.2)	932.2 (822.0–1055.3)

Values are survey-weighted mean (SD), median (IQR), or percentage unless otherwise stated. Development estimates use seven-cycle combined MEC or fasting-domain weights. The 2021–2023 estimates use phlebotomy weights for blood-analyte comparisons and fasting-subsample weights for fasting variables. No baseline-significance tests were performed.

AA = associate of arts, BMI = body mass index, CVD = cardiovascular disease, eGFR = estimated glomerular filtration rate, GED = General Educational Development, HDL = high-density lipoprotein, HDL-C = high-density lipoprotein cholesterol, IQR = interquartile range, MEC = mobile examination center, n = number, SBP = systolic blood pressure, SD = standard deviation, SIRI = systemic inflammation response index, TyG-WC = triglyceride-glucose waist circumference index, UACR = urine albumin-to-creatinine ratio.

### 3.2. Out-of-cycle performance of the core model

The primary Elastic Net core model achieved a pooled survey-weighted AUROC of 0.8490 (95% CI, 0.8410–0.8560) and PR-AUC of 0.3690 (0.3550–0.3860) (Table 2). Its Brier score was 0.0658 (0.0628–0.0687). Calibration was close to ideal, with an intercept of −0.008 (−0.077 to 0.064) and a slope of 0.995 (0.964–1.031). The corresponding XGBoost core estimates were AUROC 0.853 (0.845–0.860) and PR-AUC 0.382 (0.368–0.400).

**Table 2:** Pooled out-of-cycle performance of the locked models.

Algorithm	Model	n (events)	AUROC (95% CI)	PR-AUC (95% CI)	Brier score (95% CI)	Calibration intercept (95% CI)	Calibration slope (95% CI)
Elastic Net	Model 0 (core clinical)	37482 (4222)	0.849 (0.841 to 0.856)	0.369 (0.355 to 0.386)	0.0658 (0.0628 to 0.0687)	−0.008 (−0.077 to 0.064)	0.995 (0.964 to 1.031)
Elastic Net	Model 1 (+ renal)	37482 (4222)	0.853 (0.846 to 0.860)	0.385 (0.371 to 0.403)	0.0649 (0.0619 to 0.0679)	−0.009 (−0.077 to 0.062)	0.995 (0.963 to 1.031)
Elastic Net	Model 2 (+ TyG−WC)	15956 (1813)	0.846 (0.834 to 0.857)	0.371 (0.350 to 0.397)	0.0685 (0.0640 to 0.0727)	−0.017 (−0.130 to 0.092)	0.992 (0.945 to 1.050)
Elastic Net	Model 3 (+ SIRI)	37482 (4222)	0.850 (0.842 to 0.857)	0.372 (0.357 to 0.390)	0.0657 (0.0627 to 0.0686)	−0.007 (−0.076 to 0.067)	0.996 (0.964 to 1.033)
Elastic Net	Model 4 (combined)	15956 (1813)	0.852 (0.842 to 0.863)	0.395 (0.374 to 0.423)	0.0673 (0.0628 to 0.0716)	−0.019 (−0.122 to 0.083)	0.991 (0.948 to 1.043)
XGBoost	Model 0 (core clinical)	37482 (4222)	0.853 (0.845 to 0.860)	0.382 (0.368 to 0.400)	0.0653 (0.0623 to 0.0682)	0.050 (−0.020 to 0.121)	1.025 (0.994 to 1.060)
XGBoost	Model 1 (+ renal)	37482 (4222)	0.856 (0.848 to 0.863)	0.392 (0.378 to 0.412)	0.0646 (0.0616 to 0.0676)	0.021 (−0.052 to 0.094)	1.009 (0.978 to 1.047)
XGBoost	Model 2 (+ TyG−WC)	15956 (1813)	0.846 (0.835 to 0.858)	0.381 (0.360 to 0.410)	0.0685 (0.0638 to 0.0728)	−0.004 (−0.106 to 0.103)	0.996 (0.952 to 1.053)
XGBoost	Model 3 (+ SIRI)	37482 (4222)	0.853 (0.846 to 0.861)	0.383 (0.369 to 0.403)	0.0652 (0.0622 to 0.0681)	0.040 (−0.030 to 0.112)	1.019 (0.987 to 1.054)
XGBoost	Model 4 (combined)	15956 (1813)	0.849 (0.838 to 0.861)	0.405 (0.385 to 0.434)	0.0673 (0.0626 to 0.0717)	0.017 (−0.079 to 0.119)	1.007 (0.964 to 1.064)

Estimates are survey weighted with 95% Rao-Wu bootstrap confidence intervals. Models 2 and 4 use the fasting sample; other models use the MEC sample.

AUROC = area under the receiver operating characteristic curve, CI = confidence interval, MEC = mobile examination center, n = number, PR-AUC = area under the precision-recall curve, SIRI = systemic inflammation response index, TyG-WC = triglyceride-glucose waist circumference index.

### 3.3. Incremental value of prespecified biomarker domains

Adding eGFR and log2(UACR) to the core model improved all 4 prespecified primary metrics (Table 3, Fig. 2). For Elastic Net, the paired increases were 0.0046 (95% CI, 0.0031–0.0062) for AUROC and 0.0161 (0.0101–0.0223) for PR-AUC. Brier-score improvement was 0.0009 (0.0006–0.0012), and log-loss improvement was 0.0027 (0.0019–0.0036).

**Table 3:** Paired incremental value of the biomarker extensions.

Algorithm	Comparison	n (events)	Delta AUROC (95% CI)	Delta PR-AUC (95% CI)	Brier improvement (95% CI)	Log-loss improvement (95% CI)
Elastic Net	Renal (Model 1 vs Model 0)	37482 (4222)	0.0046 (0.0031 to 0.0062)	0.0161 (0.0101 to 0.0223)	0.0009 (0.0006 to 0.0012)	0.0027 (0.0019 to 0.0036)
Elastic Net	Metabolic (Model 2 vs paired Model 0)	15956 (1813)	0.0011 (−0.0002 to 0.0024)	0.0002 (−0.0038 to 0.0041)	0.0001 (−0.0001 to 0.0003)	0.0005 (−0.0002 to 0.0011)
Elastic Net	Inflammatory (Model 3 vs Model 0)	37482 (4222)	0.0011 (0.0004 to 0.0017)	0.0024 (−0.0004 to 0.0056)	0.0001 (0.0000 to 0.0003)	0.0005 (0.0001 to 0.0009)
Elastic Net	Combined (Model 4 vs paired Model 0)	15956 (1813)	0.0072 (0.0039 to 0.0106)	0.0241 (0.0124 to 0.0366)	0.0014 (0.0007 to 0.0020)	0.0043 (0.0024 to 0.0062)
XGBoost	Renal (Model 1 vs Model 0)	37482 (4222)	0.0029 (0.0014 to 0.0044)	0.0103 (0.0029 to 0.0183)	0.0006 (0.0003 to 0.0010)	0.0018 (0.0008 to 0.0027)
XGBoost	Metabolic (Model 2 vs paired Model 0)	15956 (1813)	0.0005 (−0.0013 to 0.0024)	−0.0067 (−0.0130 to −0.0003)	−0.0003 (−0.0007 to −0.0000)	−0.0004 (−0.0014 to 0.0005)
XGBoost	Inflammatory (Model 3 vs Model 0)	37482 (4222)	0.0008 (−0.0003 to 0.0019)	−0.0004 (−0.0047 to 0.0040)	0.0000 (−0.0002 to 0.0003)	0.0002 (−0.0005 to 0.0009)
XGBoost	Combined (Model 4 vs paired Model 0)	15956 (1813)	0.0038 (0.0004 to 0.0073)	0.0179 (0.0046 to 0.0323)	0.0009 (0.0002 to 0.0016)	0.0026 (0.0006 to 0.0047)

Positive values indicate improvement. Each extended model and core comparator use identical participants and survey weights.

AUROC = area under the receiver operating characteristic curve, CI = confidence interval, n = number, PR-AUC = area under the precision-recall curve.

**Figure 2.**
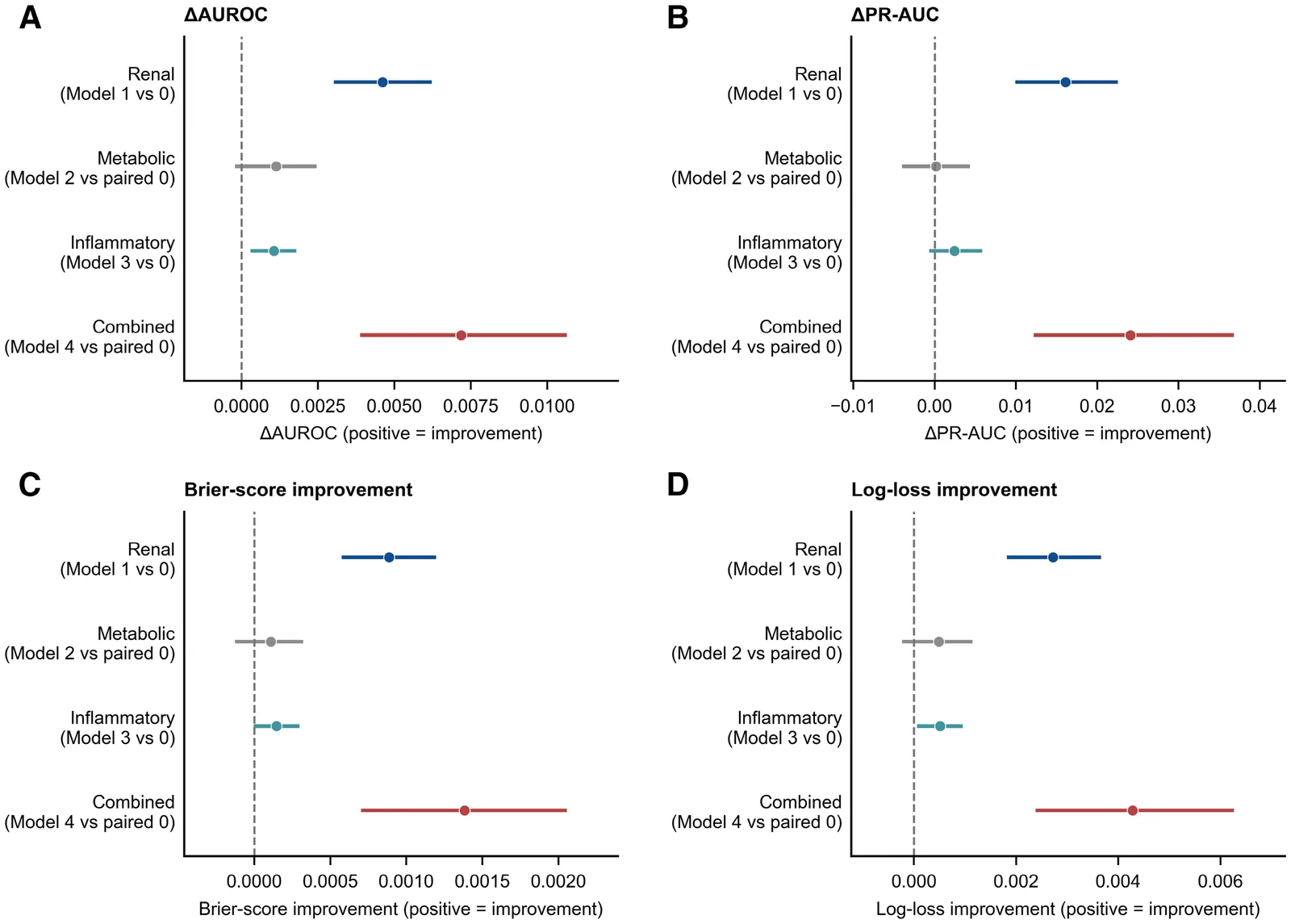
Pooled incremental value of locked biomarker models. Points are paired survey-weighted Elastic Net estimates, and horizontal lines are Rao-Wu bootstrap 95% confidence intervals. Positive values indicate improvement. Renal and inflammatory comparisons used the MEC sample; metabolic and combined comparisons used paired fasting-subsample core models.

Within the fasting sample, the combined model produced larger paired gains than TyG-WC alone. Its AUROC increased by 0.0072 (0.0039–0.0106), PR-AUC by 0.0241 (0.0124–0.0366), Brier score improved by 0.0014 (0.0007–0.0020), and log loss improved by 0.0043 (0.0024–0.0062). Fasting-domain and MEC-domain estimates describe different analysis populations, so comparisons between domains require that distinction.

TyG-WC alone showed uncertain incremental value, with 95% confidence intervals crossing zero for all 4 Elastic Net metrics. SIRI alone produced small improvements in AUROC, Brier score, and log loss, whereas its PR-AUC interval included zero. Renal markers provided the most consistent isolated-domain gains across metrics, and the combined model improved on its fasting-matched core comparator.

### 3.4. Across-cycle reproducibility and algorithm sensitivity

Random-effects pooling supported recurrence of the renal and combined effects across held-out cycles (Table 4, Fig. 3). The renal Elastic Net summary was 0.0045 (95% CI, 0.0027–0.0063; I2 = 0.0%) for AUROC and 0.0154 (0.0067–0.0240; I2 = 28.8%) for PR-AUC. The combined summaries were 0.0069 (0.0029–0.0108; I2 = 0.0%) and 0.0248 (0.0114–0.0382; I2 = 0.0%), respectively. The metabolic AUROC and PR-AUC intervals included zero, as did inflammatory PR-AUC.

**Table 4:** Random-effects summaries of cycle-specific incremental value.

Algorithm	Comparison	Delta AUROC; I2	Delta PR-AUC; I2	Brier improvement; I2	Log-loss improvement; I2
Elastic Net	Renal (Model 1 vs Model 0)	0.0045 (0.0027 to 0.0063); 0.0%	0.0154 (0.0067 to 0.0240); 28.8%	0.0008 (0.0003 to 0.0013); 44.5%	0.0026 (0.0013 to 0.0039); 31.9%
Elastic Net	Metabolic (Model 2 vs paired Model 0)	0.0013 (−0.0000 to 0.0027); 0.0%	0.0009 (−0.0033 to 0.0052); 0.0%	0.0002 (−0.0000 to 0.0004); 0.0%	0.0007 (0.0001 to 0.0014); 0.0%
Elastic Net	Inflammatory (Model 3 vs Model 0)	0.0011 (0.0002 to 0.0020); 20.3%	0.0037 (−0.0003 to 0.0078); 25.5%	0.0002 (−0.0000 to 0.0003); 25.6%	0.0006 (0.0001 to 0.0011); 1.3%
Elastic Net	Combined (Model 4 vs paired Model 0)	0.0069 (0.0029 to 0.0108); 0.0%	0.0248 (0.0114 to 0.0382); 0.0%	0.0013 (0.0005 to 0.0020); 0.0%	0.0038 (0.0018 to 0.0058); 0.0%
XGBoost	Renal (Model 1 vs Model 0)	0.0026 (0.0008 to 0.0044); 0.0%	0.0083 (−0.0008 to 0.0175); 0.0%	0.0005 (0.0001 to 0.0010); 22.9%	0.0014 (0.0003 to 0.0025); 0.0%
XGBoost	Metabolic (Model 2 vs paired Model 0)	0.0008 (−0.0021 to 0.0038); 42.0%	−0.0023 (−0.0119 to 0.0073); 52.4%	−0.0002 (−0.0007 to 0.0004); 48.9%	−0.0001 (−0.0018 to 0.0016); 54.2%
XGBoost	Inflammatory (Model 3 vs Model 0)	0.0008 (−0.0006 to 0.0022); 0.0%	0.0026 (−0.0027 to 0.0078); 28.6%	0.0002 (−0.0000 to 0.0003); 0.0%	0.0005 (−0.0001 to 0.0010); 0.0%
XGBoost	Combined (Model 4 vs paired Model 0)	0.0031 (−0.0033 to 0.0096); 67.3%	0.0190 (−0.0046 to 0.0426); 53.1%	0.0009 (−0.0003 to 0.0021); 48.4%	0.0024 (−0.0017 to 0.0064); 65.6%

Effects were pooled across 7 held-out cycles using restricted maximum likelihood and modified Hartung-Knapp 95% confidence intervals. I2 quantifies between-cycle heterogeneity.

AUROC = area under the receiver operating characteristic curve, I2 = percentage of total variability attributed to between-cycle heterogeneity, PR-AUC = area under the precision-recall curve.

**Figure 3.**
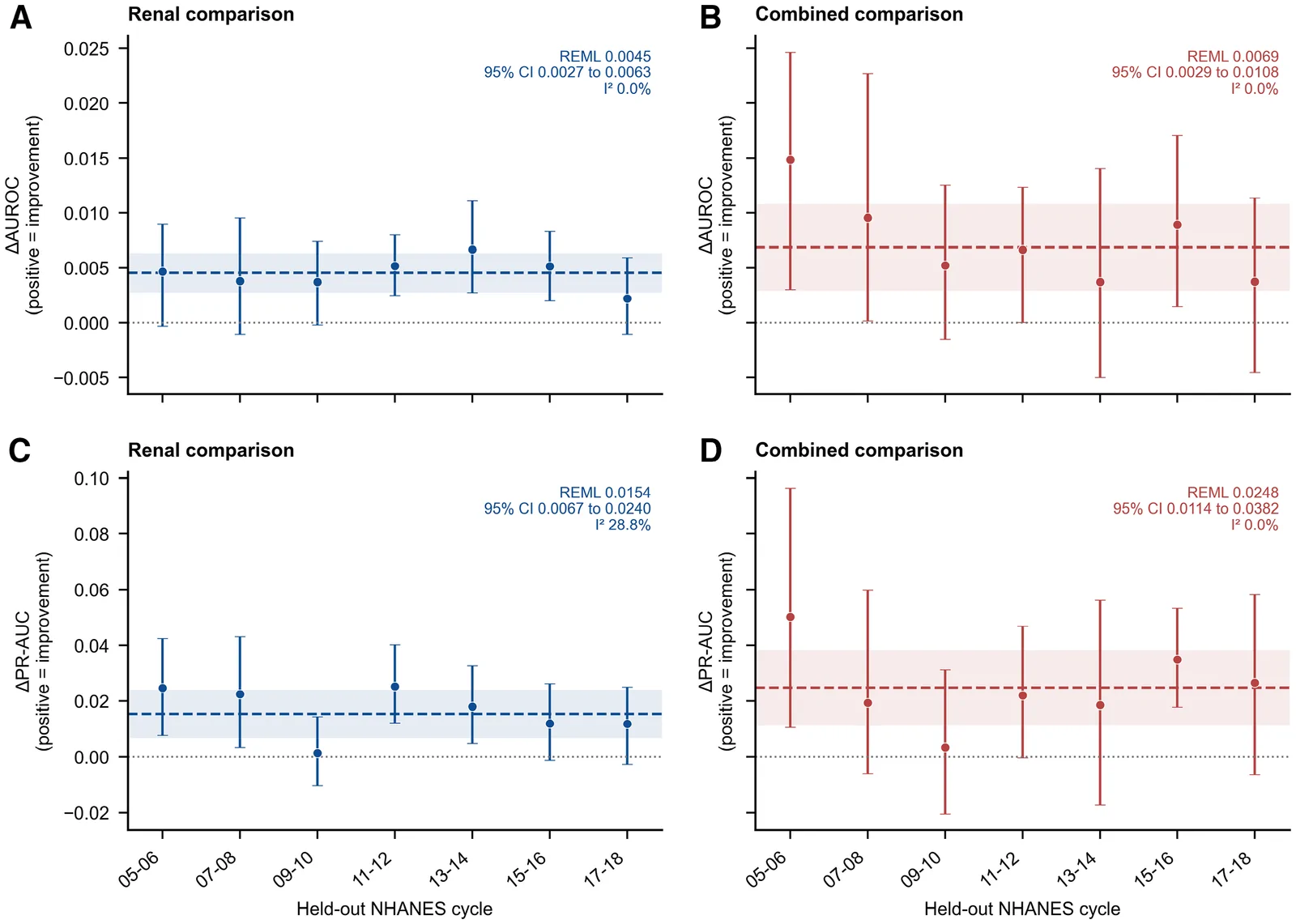
Across-cycle reproducibility of renal and combined gains. Points show cycle-specific survey-bootstrap estimates and vertical 95% confidence intervals. Dashed lines and shaded bands show restricted maximum-likelihood random-effects summaries with modified Hartung-Knapp 95% confidence intervals. Panels assess recurrence across held-out survey cycles. Y-axis limits are shared between the renal and combined panels within each metric row; AUROC and PR-AUC rows use different scales because the metrics have different numerical ranges.

Pooled paired XGBoost analyses also showed positive renal and combined changes, whereas cycle-level random-effects estimates were less consistent across metrics (Tables 3 and 4). For the combined XGBoost comparison, I2 ranged from 48.4 to 67.3% across the 4 metrics, and each modified Hartung-Knapp interval included zero. These results distinguish positive pooled increments from their less certain reproducibility across cycles.

### 3.5. Postaudit corrected temporal assessment in NHANES 2021–2023

The postaudit corrected temporal phlebotomy sample included 5755 adults and 739 CVD events; the fasting sample included 2940 adults and 355 events (Table 5). The Elastic Net phlebotomy core achieved AUROC 0.8533 (0.8313–0.8753), PR-AUC 0.3979 (0.3622–0.4384), Brier score 0.0707 (0.0649–0.0770), and log loss 0.2392 (0.2226–0.2569). Its calibration intercept was −0.0805 (−0.2755 to 0.1327), slope 0.9904 (0.8919–1.1152), and observed-to-expected ratio 0.9536 (0.8898–1.0171).

**Table 5:** Postaudit corrected temporal incremental value in the 2021–2023 National Health and Nutrition Examination Survey.

Algorithm	Comparison	n (events)	Delta AUROC (95% CI)	Delta PR-AUC (95% CI)	Brier improvement (95% CI)	Log-loss improvement (95% CI)
Elastic Net	Renal	5755 (739)	0.0041 (0.0001 to 0.0081)	0.0217 (0.0093 to 0.0310)	0.0010 (0.0003 to 0.0017)	0.0029 (0.0006 to 0.0053)
Elastic Net	Metabolic	2940 (355)	0.0010 (−0.0023 to 0.0039)	0.0023 (−0.0073 to 0.0121)	0.0000 (−0.0004 to 0.0004)	0.0005 (−0.0011 to 0.0019)
Elastic Net	Inflammatory	5755 (739)	0.0015 (0.0001 to 0.0030)	0.0088 (−0.0026 to 0.0189)	0.0004 (−0.0001 to 0.0009)	0.0012 (0.0000 to 0.0022)
Elastic Net	Combined	2940 (355)	0.0074 (0.0016 to 0.0124)	0.0250 (0.0040 to 0.0418)	0.0014 (0.0001 to 0.0025)	0.0050 (0.0014 to 0.0082)
XGBoost	Renal	5755 (739)	0.0022 (−0.0016 to 0.0066)	0.0080 (−0.0071 to 0.0213)	0.0005 (−0.0003 to 0.0013)	0.0014 (−0.0011 to 0.0040)
XGBoost	Metabolic	2940 (355)	−0.0010 (−0.0046 to 0.0024)	−0.0068 (−0.0257 to 0.0125)	−0.0005 (−0.0012 to 0.0002)	−0.0011 (−0.0026 to 0.0003)
XGBoost	Inflammatory	5755 (739)	0.0017 (−0.0013 to 0.0051)	0.0073 (−0.0046 to 0.0214)	0.0005 (0.0000 to 0.0012)	0.0014 (−0.0003 to 0.0034)
XGBoost	Combined	2940 (355)	0.0035 (−0.0032 to 0.0104)	0.0105 (−0.0120 to 0.0352)	0.0006 (−0.0008 to 0.0020)	0.0024 (−0.0015 to 0.0064)

Frozen pipelines were applied without refitting or recalibration. Positive values indicate improvement. Confidence intervals use 2000 Rao-Wu rescaled bootstrap replicates.

AUROC = area under the receiver operating characteristic curve, CI = confidence interval, n = number, PR-AUC = area under the precision-recall curve.

The renal Elastic Net extension improved all 4 paired metrics: AUROC 0.0041 (0.0001–0.0081), PR-AUC 0.0217 (0.0093–0.0310), Brier score 0.0010 (0.0003–0.0017), and log loss 0.0029 (0.0006–0.0053). The combined extension changed AUROC by 0.0074 (0.0016–0.0124), PR-AUC by 0.0250 (0.0040–0.0418), Brier score by 0.0014 (0.0001–0.0025), and log loss by 0.0050 (0.0014–0.0082). Metabolic-only AUROC was 0.0010 (-0.0023 to 0.0039); inflammatory AUROC was 0.0015 (0.0001–0.0030), but its PR-AUC and Brier intervals crossed zero. For transparency, the initial unmatched fasting-core run and both correction stages are reported in Table S12, Supplemental Digital Content 8. For the combined Elastic Net comparison, the original AUROC increment was 0.0056 (95% CI, −0.0009 to 0.0115), compared with the final corrected estimate of 0.0074 (0.0016–0.0124).

XGBoost core discrimination was similar or slightly higher, but no biomarker extension improved all 4 temporal metrics with confidence intervals excluding zero. The combined XGBoost AUROC increment was 0.0035 (95% CI, −0.0032 to 0.0104); its PR-AUC, Brier-score, and log-loss intervals also crossed zero. Temporal improvement across all 4 metrics was confined to the renal and combined Elastic Net comparisons.

Grouped survey-weighted calibration broadly tracked the ideal line for the corrected Elastic Net models (Fig. 4). The core calibration intercept was −0.0805, slope 0.9904, and observed-to-expected ratio 0.9536; the renal values were −0.0769, 0.9900, and 0.9573. The combined fasting model values were −0.1623, 0.9629, and 0.9274. Full estimates are in Table S6, Supplemental Digital Content 9. No temporal recalibration was performed.

**Figure 4.**
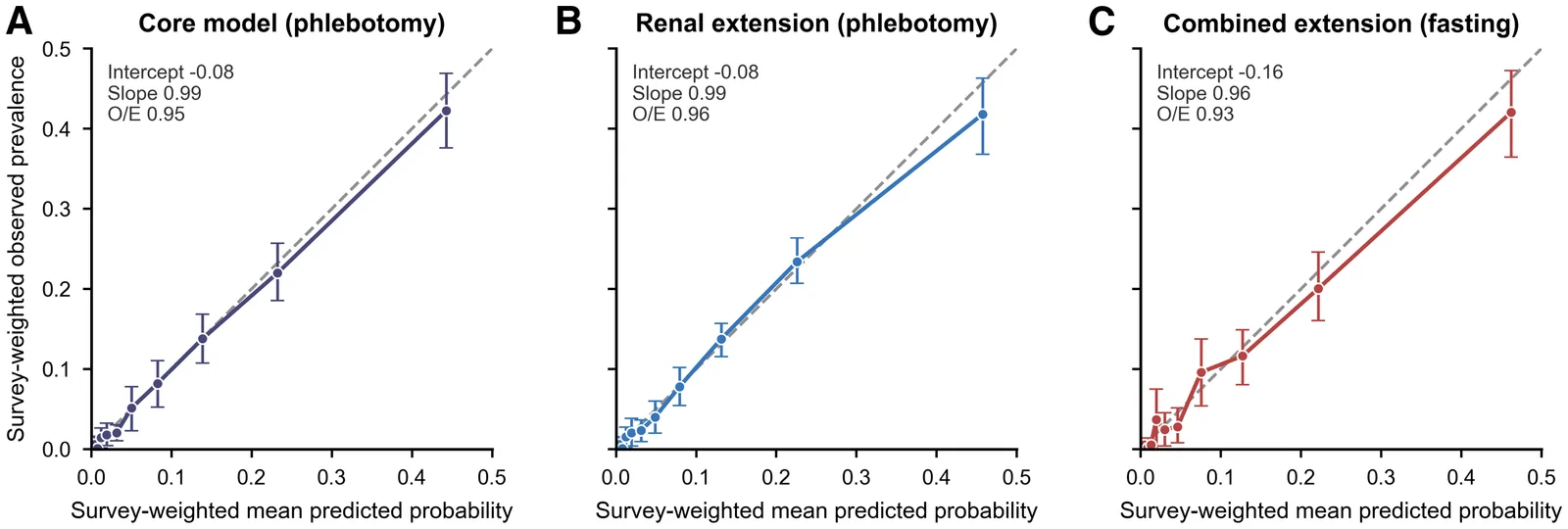
Postaudit corrected temporal calibration of Elastic Net models. Points show survey-weighted observed CVD prevalence within fixed survey-weighted deciles of predicted probability; vertical bars are 95% percentile intervals from 2000 Rao-Wu rescaled bootstrap replicates. Dashed lines show ideal calibration. Intercept, slope, and observed-to-expected ratio are model-level estimates. No temporal recalibration was performed.

### 3.6. Missing-data and class-imbalance sensitivities

The direction of renal and combined discrimination gains was preserved across the main fold-median UACR approach, complete-case refitting, nested multiple imputation, and stabilized inverse-probability weighting (Table S1, Supplemental Digital Content 10 and Figure S1, Supplemental Digital Content 11). Across cycles, median renal AUROC changes ranged from 0.0043 to 0.0050 across these methods, and median combined changes ranged from 0.0063 to 0.0069. Class-weighted Elastic Net refits likewise retained positive median renal and combined discrimination changes, whereas metabolic and inflammatory effects remained small or variable (Supplemental Table S2, Supplemental Digital Content 12). These analyses assessed the robustness of direction using the prespecified sensitivity approaches. In the strict complete-negative outcome sensitivity, the unchanged renal Elastic Net predictions yielded an AUROC increment of 0.0046 (0.0030–0.0062) in development out-of-cycle evaluation and 0.0041 (−0.0001 to 0.0081) in 2021 to 2023 (Table S8, Supplemental Digital Content 13).

### 3.7. Exploratory decision-curve analysis

Net-benefit differences varied across threshold probabilities (Fig. 5; Supplemental Table S3, Supplemental Digital Content 14). The renal Elastic Net extension exceeded its paired core model at 29 of 30 thresholds and had a confidence interval above zero at 16 thresholds. The combined extension exceeded its core comparator at 26 thresholds and had a confidence interval above zero at 12 thresholds. Maximum estimated net-benefit differences were modest: 0.0021 at a threshold of 0.29 for the renal model and 0.0043 at 0.30 for the combined model. These threshold-specific estimates were exploratory, with pointwise intervals unadjusted for the 30 threshold comparisons.

**Figure 5.**
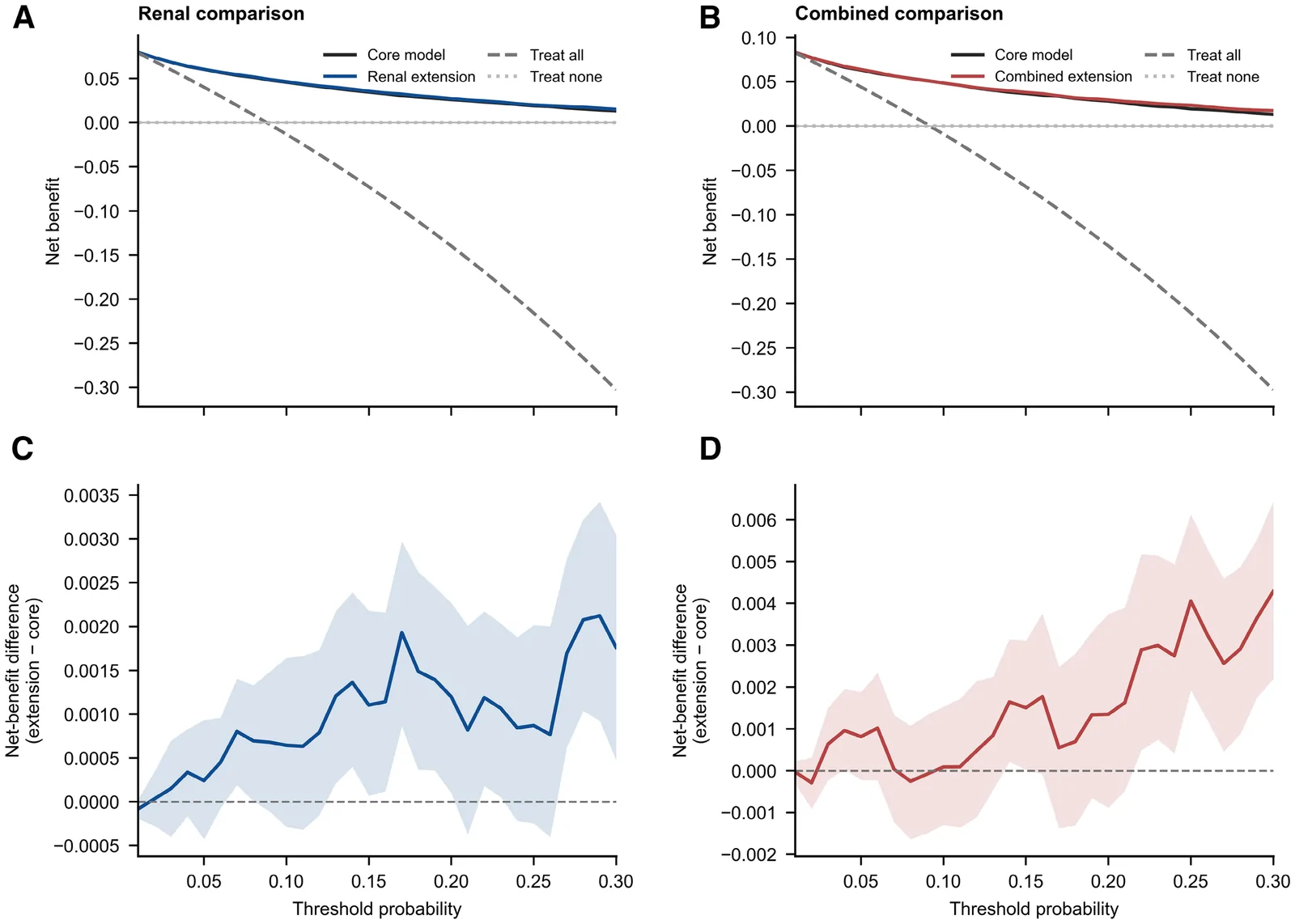
Exploratory decision curves for renal and combined extensions. Using pooled 2005–2018 out-of-cycle predictions, upper panels compare core, extended, treat-all, and treat-none strategies. Lower panels show extended-minus-core net benefit with pointwise Rao-Wu bootstrap 95% confidence bands. Estimates describe threshold-specific classification of prevalent CVD, with no prespecified clinical action attached to the thresholds.

## 4. Discussion

Renal markers provided modest incremental information for identifying prevalent self-reported CVD, with more consistent gains under Elastic Net than XGBoost. The combined extension improved discrimination relative to its fasting-matched core, while TyG-WC alone and SIRI contributed less consistent information. The corrected 2021 to 2023 assessment supported the direction of the renal and combined Elastic Net findings.

The effect size is central to interpretation. Renal AUROC increased by 0.0046 in development and 0.0041 in the corrected temporal assessment, whose lower 95% confidence limit was 0.0001. These increments and the accompanying PR-AUC changes were small in absolute terms. Improvements in Brier score and log loss were consistent with the discrimination findings, while decision-curve net-benefit differences were also modest. Clinical utility remains an open question because the analysis classified prevalent disease and evaluated thresholds without a specified clinical action. Prospective evaluation should link biomarker use to a defined decision, its consequences, and patient outcomes.

The renal findings align with longitudinal evidence linking eGFR and UACR to cardiovascular outcomes beyond traditional factors.^[1–3]^ In this study, however, biomarker measurements and self-reported disease history were assessed cross-sectionally, leaving their temporal ordering unresolved. Reduced eGFR, elevated UACR, and increased SIRI may reflect consequences of established CVD, its treatment, or shared disease processes, as well as antecedent abnormalities. This makes reverse causation a substantive alternative explanation for the observed associations. UACR was measured from 1 spot urine specimen, whereas persistent albuminuria requires confirmation with repeat assessment.^[22]^ The analysis therefore describes biomarker information associated with existing disease burden.

Composite blood-cell indices provide context for the limited incremental contribution of SIRI. The systemic immune-inflammation index (SII) combines platelet count with the neutrophil-to-lymphocyte ratio, whereas SIRI uses monocyte count in place of platelets. Hayıroğlu and colleagues associated higher SII with long-term mortality and appropriate defibrillator therapy in patients with heart failure with reduced ejection fraction.^[23]^ Tezen and colleagues associated higher SII with contrast-induced nephropathy after coronary angiography in patients with non-ST-segment elevation myocardial infarction.^[24]^ These studies illustrate potential prognostic applications of cellular inflammatory indices in selected cardiac populations. Their outcomes and clinical settings differ from population-level classification of prevalent CVD, and the 2 indices capture partly different cellular components. Our small SIRI increments suggest that association with inflammatory burden can coexist with limited additional discrimination beyond routine clinical factors.

The algorithm comparison is important for interpreting reproducibility. XGBoost showed positive pooled renal and combined increments during development, but the combined random-effects intervals and all 4 combined temporal intervals included zero. Similar or slightly higher XGBoost core discrimination may also leave limited room for additional separation. Differences in regularization, functional form, and variance may influence the stability of such small increments. The findings are therefore algorithm-dependent, with the clearest cross-cycle and later-period consistency observed for Elastic Net.

NHANES 2021–2023 differed from earlier cycles in sample design, fieldwork conditions, and response rates. NCHS reported interview and examination response rates of 34.6 and 25.7%, compared with 51.0 and 46.9% in 2017–2018.^[13]^ This shift tests transport across survey periods while reducing direct comparability. The corrected temporal analysis additionally followed inspection of the initial outputs, as detailed in Methods and Table S12, Supplemental Digital Content 8. Its contribution is supportive, with primary evidence provided by the prespecified development-period cycle validation.

Strengths include a fixed clinical comparator, training-only preprocessing and tuning, complete hyperparameter grids, same-sample and same-weight incremental contrasts, entire-cycle holdout, survey-aware bootstrap intervals, and missing-UACR and class-imbalance sensitivities. Cycle-level meta-analysis showed whether effects recurred rather than relying only on a pooled split. The public archive preserves code, configurations, frozen artifacts, aggregate results, and both correction audits.

Several limitations shape interpretation. Self-report agreement differs by cardiovascular component, particularly for heart failure; combining these conditions may dilute component-specific associations.^[17]^ Cross-sectional ascertainment leaves reverse causation and treatment effects unresolved, and a single UACR measurement may misclassify persistent albuminuria. Fasting analyses were smaller, and validation was restricted to NHANES. Survey weights entered model fitting as case weights, with design-based inference applied to performance estimates. Bootstrap intervals condition on fixed predictions and exclude the variability of repeating preprocessing, tuning, and fitting. Multiple models, algorithms, metrics, sensitivities, and decision thresholds were evaluated without multiplicity correction, so estimates are interpreted as supportive rather than family-wise confirmatory evidence. The postaudit temporal assessment and survey-period changes further qualify transportability. Independent prospective studies with adjudicated outcomes would address temporality, generalizability, and clinical decision value.

## 5. Conclusions

Renal and combined biomarker extensions yielded modest, algorithm-dependent gains in classifying prevalent self-reported CVD across NHANES cycles. Gains were more consistent with Elastic Net, including in the corrected temporal assessment. The findings characterize cross-sectional association patterns and motivate prospective evaluation of renal markers alongside routine clinical factors. Clinical application requires independent studies with adjudicated outcomes, defined decision pathways, and assessment of patient benefit.

## Author contributions

**Conceptualization:** Haoyang Li.

**Data curation:** Haoyang Li.

**Formal analysis:** Haoyang Li.

**Methodology:** Haoyang Li, Xiang Li.

**Software:** Haoyang Li.

**Supervision:** Xuefeng Shi.

**Validation:** Haoyang Li, Xiang Li.

**Visualization:** Haoyang Li.

**Writing – original draft:** Haoyang Li.

**Writing – review and editing:** Xiang Li, Xuefeng Shi.




























