# Supplementary material for: Incremental value of renal, metabolic, and inflammatory markers for prevalent self-reported cardiovascular disease across NHANES cycles: A repeated cross-sectional validation study
Source: Medicine (Baltimore). 2026 Nov 6;105(45):e51425. doi: 10.1097/MD.0000000000051425 (PMC13747496; doi:10.1097/MD.0000000000051425)
Supplement: Supplementary file 1 [file medi-105-e51425-s001.docx]

**Supplemental Table S9**

Incremental value of renal, metabolic, and inflammatory markers for prevalent self-reported cardiovascular disease across NHANES cycles: a repeated cross-sectional validation study

First author: Haoyang Li; second author: Xiang Li

# Table S9. NHANES variable dictionary and cycle harmonization

| **role** | **analytic_name** | **domain** | **raw_NHANES_variables** | **units** | **coding_or_formula** | **cycle_harmonization** | **sample_or_weight_requirement** | **missing_handling** | **notes** |
| --- | --- | --- | --- | --- | --- | --- | --- | --- | --- |
| predictor | age | core clinical | RIDAGEYR | years | RIDAGEYR | 2005-2006:Y; 2007-2008:Y; 2009-2010:Y; 2011-2012:Y; 2013-2014:Y; 2015-2016:Y; 2017-2018:Y; 2021-2023:Y | 2005-2018 MEC domain (WTMEC2YR); 2021-2023 phlebotomy domain (WTPH2YR); fasting weight for Models 2 and 4 | training-fold median | LOCKED_STAGE2 |
| predictor | sex | core clinical | RIAGENDR | category | categorical recode | 2005-2006:Y; 2007-2008:Y; 2009-2010:Y; 2011-2012:Y; 2013-2014:Y; 2015-2016:Y; 2017-2018:Y; 2021-2023:Y | 2005-2018 MEC domain (WTMEC2YR); 2021-2023 phlebotomy domain (WTPH2YR); fasting weight for Models 2 and 4 | training-fold explicit Missing category | LOCKED_STAGE2 |
| predictor | race_ethnicity | core clinical | RIDRETH3 or RIDRETH1 | category | categorical recode | 2005-2006:Y; 2007-2008:Y; 2009-2010:Y; 2011-2012:Y; 2013-2014:Y; 2015-2016:Y; 2017-2018:Y; 2021-2023:Y | 2005-2018 MEC domain (WTMEC2YR); 2021-2023 phlebotomy domain (WTPH2YR); fasting weight for Models 2 and 4 | training-fold explicit Missing category | LOCKED_STAGE2 |
| predictor | education | core clinical | DMDEDUC2 | category | five education categories (codes 1-5); special codes treated as missing | 2005-2006:Y; 2007-2008:Y; 2009-2010:Y; 2011-2012:Y; 2013-2014:Y; 2015-2016:Y; 2017-2018:Y; 2021-2023:Y | 2005-2018 MEC domain (WTMEC2YR); 2021-2023 phlebotomy domain (WTPH2YR); fasting weight for Models 2 and 4 | training-fold explicit Missing category | Education was retained in the main clinical core; PIR was excluded from the main models. |
| predictor | smoking | core clinical | SMQ020; SMQ040 | category | never/former/current recode | 2005-2006:Y; 2007-2008:Y; 2009-2010:Y; 2011-2012:Y; 2013-2014:Y; 2015-2016:Y; 2017-2018:Y; 2021-2023:Y | 2005-2018 MEC domain (WTMEC2YR); 2021-2023 phlebotomy domain (WTPH2YR); fasting weight for Models 2 and 4 | training-fold explicit Missing category | LOCKED_STAGE2 |
| predictor | bmi | core clinical | BMXBMI | kg/m2 | body mass index | 2005-2006:Y; 2007-2008:Y; 2009-2010:Y; 2011-2012:Y; 2013-2014:Y; 2015-2016:Y; 2017-2018:Y; 2021-2023:Y | 2005-2018 MEC domain (WTMEC2YR); 2021-2023 phlebotomy domain (WTPH2YR); fasting weight for Models 2 and 4 | training-fold median | BMI was retained in the main clinical core; waist circumference entered the TyG-WC extension. |
| predictor | mean_sbp | core clinical | BPXSY1-BPXSY4 or 2021-2023 BPXO equivalents | mmHg | mean of valid SBP readings | 2005-2006:Y; 2007-2008:Y; 2009-2010:Y; 2011-2012:Y; 2013-2014:Y; 2015-2016:Y; 2017-2018:Y; 2021-2023:Y | 2005-2018 MEC domain (WTMEC2YR); 2021-2023 phlebotomy domain (WTPH2YR); fasting weight for Models 2 and 4 | training-fold median | LOCKED_STAGE2 |
| predictor | diabetes | core clinical | DIQ010; LBXGH; LBXGLU | binary | self-reported diabetes (DIQ010=1), glycated hemoglobin >=6.5%, or fasting glucose >=126 mg/dL; missing if all sources missing | 2005-2006:Y; 2007-2008:Y; 2009-2010:Y; 2011-2012:Y; 2013-2014:Y; 2015-2016:Y; 2017-2018:Y; 2021-2023:Y | 2005-2018 MEC domain (WTMEC2YR); 2021-2023 phlebotomy domain (WTPH2YR); fasting weight for Models 2 and 4 | training-fold explicit Missing category | LOCKED_STAGE2 |
| predictor | total_cholesterol | core clinical | LBXTC | mg/dL | LBXTC | 2005-2006:Y; 2007-2008:Y; 2009-2010:Y; 2011-2012:Y; 2013-2014:Y; 2015-2016:Y; 2017-2018:Y; 2021-2023:Y | 2005-2018 MEC domain (WTMEC2YR); 2021-2023 phlebotomy domain (WTPH2YR); fasting weight for Models 2 and 4 | training-fold median | LOCKED_STAGE2 |
| predictor | hdl_cholesterol | core clinical | LBDHDD or LBDHDL or LBXHDD | mg/dL | available HDL-C variable | 2005-2006:Y; 2007-2008:Y; 2009-2010:Y; 2011-2012:Y; 2013-2014:Y; 2015-2016:Y; 2017-2018:Y; 2021-2023:Y | 2005-2018 MEC domain (WTMEC2YR); 2021-2023 phlebotomy domain (WTPH2YR); fasting weight for Models 2 and 4 | training-fold median | LOCKED_STAGE2 |
| predictor | egfr | renal | LBXSCR; RIDAGEYR; RIAGENDR | mL/min/1.73 m2 | 142*min(Scr/k,1)^alpha*max(Scr/k,1)^-1.2*0.9938^age*(1.012 if female) | 2005-2006:Y; 2007-2008:Y; 2009-2010:Y; 2011-2012:Y; 2013-2014:Y; 2015-2016:Y; 2017-2018:Y; 2021-2023:Y | 2005-2018 MEC domain (WTMEC2YR); 2021-2023 phlebotomy domain (WTPH2YR); fasting weight for Models 2 and 4 | training-fold median | Require Scr>0, age non-missing, sex non-missing; no race coefficient. |
| predictor | log2_uacr | renal | URDACT or URXUMA and URXUCR | per doubling of mg/g | log2(UACR) | 2005-2006:Y; 2007-2008:Y; 2009-2010:Y; 2011-2012:Y; 2013-2014:Y; 2015-2016:Y; 2017-2018:Y; 2021-2023:Y | 2005-2018 MEC domain (WTMEC2YR); 2021-2023 phlebotomy domain (WTPH2YR); fasting weight for Models 2 and 4 | training-fold median | Allowed only because audited valid UACR values are >0. |
| predictor | tyg_wc | metabolic | LBXTR or LBXTLG; LBXGLU; BMXWAIST | index-cm | ln[TG*glucose/2] * waist(cm) | 2005-2006:Y; 2007-2008:Y; 2009-2010:Y; 2011-2012:Y; 2013-2014:Y; 2015-2016:Y; 2017-2018:Y; 2021-2023:Y | fasting subsample; WTSAF2YR | training-fold median | Fasting-core sample; no outcome-driven cutoff selection. |
| predictor | siri | inflammatory | neutrophil; monocyte; lymphocyte absolute counts or WBC percentages | derived hematologic index | neutrophil_abs * monocyte_abs / lymphocyte_abs | 2005-2006:Y; 2007-2008:Y; 2009-2010:Y; 2011-2012:Y; 2013-2014:Y; 2015-2016:Y; 2017-2018:Y; 2021-2023:Y | 2005-2018 MEC domain (WTMEC2YR); 2021-2023 phlebotomy domain (WTPH2YR); fasting weight for Models 2 and 4 | training-fold median | Require lymphocyte_abs>0; use absolute counts; if absolute count missing, derive from WBC and percentages only as documented. |
| outcome component | congestive_heart_failure | self-reported prevalent CVD | MCQ160B | binary | NHANES yes=1, no=2; special missing codes excluded | 2005-2006:Y; 2007-2008:Y; 2009-2010:Y; 2011-2012:Y; 2013-2014:Y; 2015-2016:Y; 2017-2018:Y; 2021-2023:Y | observed component response | special responses treated as missing | Component of the prevalent self-reported CVD outcome; excluded from predictor sets. |
| outcome component | coronary_heart_disease | self-reported prevalent CVD | MCQ160C | binary | NHANES yes=1, no=2; special missing codes excluded | 2005-2006:Y; 2007-2008:Y; 2009-2010:Y; 2011-2012:Y; 2013-2014:Y; 2015-2016:Y; 2017-2018:Y; 2021-2023:Y | observed component response | special responses treated as missing | Component of the prevalent self-reported CVD outcome; excluded from predictor sets. |
| outcome component | angina | self-reported prevalent CVD | MCQ160D | binary | NHANES yes=1, no=2; special missing codes excluded | 2005-2006:Y; 2007-2008:Y; 2009-2010:Y; 2011-2012:Y; 2013-2014:Y; 2015-2016:Y; 2017-2018:Y; 2021-2023:Y | observed component response | special responses treated as missing | Component of the prevalent self-reported CVD outcome; excluded from predictor sets. |
| outcome component | heart_attack_or_myocardial_infarction | self-reported prevalent CVD | MCQ160E | binary | NHANES yes=1, no=2; special missing codes excluded | 2005-2006:Y; 2007-2008:Y; 2009-2010:Y; 2011-2012:Y; 2013-2014:Y; 2015-2016:Y; 2017-2018:Y; 2021-2023:Y | observed component response | special responses treated as missing | Component of the prevalent self-reported CVD outcome; excluded from predictor sets. |
| outcome component | stroke | self-reported prevalent CVD | MCQ160F | binary | NHANES yes=1, no=2; special missing codes excluded | 2005-2006:Y; 2007-2008:Y; 2009-2010:Y; 2011-2012:Y; 2013-2014:Y; 2015-2016:Y; 2017-2018:Y; 2021-2023:Y | observed component response | special responses treated as missing | Component of the prevalent self-reported CVD outcome; excluded from predictor sets. |
| composite outcome | cvd | self-reported prevalent CVD | MCQ160B; MCQ160C; MCQ160D; MCQ160E; MCQ160F | binary | 1 if any observed component is positive; 0 if no observed component is positive and at least one component is observed; missing if all five are missing | same five questions in every included cycle | adult nonpregnant analytic population | strict sensitivity required all five components observed for classification as a noncase | Outcome components were excluded from every predictor set. |
| survey design | combined_mec_weight | survey design | WTMEC2YR | weight | 2005-2018 cycle-specific MEC weights divided by seven | 2005-2018 development cycles | MEC or fasting domain as specified | analysis weights <=1e-8 excluded, including SAS special-missing sentinels represented as tiny positive values; valid strata and PSU required | Rao-Wu rescaled bootstrap respected cycle-specific strata and PSUs. |
| survey design | temporal_phlebotomy_weight | survey design | WTPH2YR | weight | 2021-2023 phlebotomy weight for Models 0, 1, and 3; no divisor | 2021-2023 only | MEC or fasting domain as specified | analysis weights <=1e-8 excluded, including SAS special-missing sentinels represented as tiny positive values; valid strata and PSU required | Rao-Wu rescaled bootstrap respected cycle-specific strata and PSUs. |
| survey design | combined_fasting_weight | survey design | WTSAF2YR | weight | cycle-specific fasting-subsample weights divided by number of pooled cycles within period | available in every included cycle | MEC or fasting domain as specified | analysis weights <=1e-8 excluded, including SAS special-missing sentinels represented as tiny positive values; valid strata and PSU required | Rao-Wu rescaled bootstrap respected cycle-specific strata and PSUs. |
| survey design | strata | survey design | SDMVSTRA | identifier | masked variance stratum; cycle included in the design key | available in every included cycle | MEC or fasting domain as specified | analysis weights <=1e-8 excluded, including SAS special-missing sentinels represented as tiny positive values; valid strata and PSU required | Rao-Wu rescaled bootstrap respected cycle-specific strata and PSUs. |
| survey design | psu | survey design | SDMVPSU | identifier | masked variance PSU; cycle included in the design key | available in every included cycle | MEC or fasting domain as specified | analysis weights <=1e-8 excluded, including SAS special-missing sentinels represented as tiny positive values; valid strata and PSU required | Rao-Wu rescaled bootstrap respected cycle-specific strata and PSUs. |

Abbreviations. AUROC, area under the receiver operating characteristic curve; CI, confidence interval; CVD, cardiovascular disease; DCA, decision-curve analysis; eGFR, estimated glomerular filtration rate; I2, percentage of total variability attributed to between-cycle heterogeneity; MEC, mobile examination center; MI, multiple imputation; n, number; PR-AUC, area under the precision-recall curve; SIRI, systemic inflammation response index; TyG-WC, triglyceride-glucose waist circumference index; UACR, urine albumin-to-creatinine ratio. NHANES, National Health and Nutrition Examination Survey; BMI, body mass index; SBP, systolic blood pressure; HDL-C, high-density lipoprotein cholesterol; PIR, poverty-income ratio; PSU, primary sampling unit; TG, triglycerides; WBC, white blood cell count; SAS, Statistical Analysis System. Uppercase raw-variable identifiers are NHANES codebook field names.
