# Supplementary material for: Incremental value of renal, metabolic, and inflammatory markers for prevalent self-reported cardiovascular disease across NHANES cycles: A repeated cross-sectional validation study
Source: Medicine (Baltimore). 2026 Nov 6;105(45):e51425. doi: 10.1097/MD.0000000000051425 (PMC13747496; doi:10.1097/MD.0000000000051425)
Supplement: Supplementary file 2 [file medi-105-e51425-s002.docx]

**Supplemental Table S11**

Incremental value of renal, metabolic, and inflammatory markers for prevalent self-reported cardiovascular disease across NHANES cycles: a repeated cross-sectional validation study

First author: Haoyang Li; second author: Xiang Li

# Table S11. Coefficients of the final Elastic Net models

| **model** | **term** | **source_predictor** | **coefficient** | **coefficient_scale** | **exp(coefficient)** |
| --- | --- | --- | --- | --- | --- |
| model0_core | intercept | intercept | -1.221483576388111 | model log-odds intercept after preprocessing | 0.2947924951593318 |
| model0_core | age | age | 1.3959867453587385 | one training-sample standard deviation after median imputation | 4.038958030070232 |
| model0_core | bmi | bmi | 0.1940810674793806 | one training-sample standard deviation after median imputation | 1.2141947106712072 |
| model0_core | mean_sbp | mean_sbp | -0.0532401102444249 | one training-sample standard deviation after median imputation | 0.9481523240619484 |
| model0_core | total_cholesterol | total_cholesterol | -0.3278462828142051 | one training-sample standard deviation after median imputation | 0.7204737603925965 |
| model0_core | hdl_cholesterol | hdl_cholesterol | -0.1172273739969801 | one training-sample standard deviation after median imputation | 0.889382947615948 |
| model0_core | sex_Female | sex | -0.6826456440198413 | one-hot indicator with all levels retained (drop=None); penalized joint-model coefficient, not an unadjusted reference-category contrast | 0.5052784356024967 |
| model0_core | sex_Male | sex | -0.5177431328485443 | one-hot indicator with all levels retained (drop=None); penalized joint-model coefficient, not an unadjusted reference-category contrast | 0.5958638170855887 |
| model0_core | race_ethnicity_Mexican American | race_ethnicity | -0.505010062231698 | one-hot indicator with all levels retained (drop=None); penalized joint-model coefficient, not an unadjusted reference-category contrast | 0.6034995028446615 |
| model0_core | race_ethnicity_Non-Hispanic Asian | race_ethnicity | -0.5355025837743405 | one-hot indicator with all levels retained (drop=None); penalized joint-model coefficient, not an unadjusted reference-category contrast | 0.5853750162172824 |
| model0_core | race_ethnicity_Non-Hispanic Black | race_ethnicity | 0.0067253401307593 | one-hot indicator with all levels retained (drop=None); penalized joint-model coefficient, not an unadjusted reference-category contrast | 1.0067480060141314 |
| model0_core | race_ethnicity_Non-Hispanic White | race_ethnicity | -0.1156112966559091 | one-hot indicator with all levels retained (drop=None); penalized joint-model coefficient, not an unadjusted reference-category contrast | 0.8908214212743951 |
| model0_core | race_ethnicity_Other Hispanic | race_ethnicity | -0.2937602687162095 | one-hot indicator with all levels retained (drop=None); penalized joint-model coefficient, not an unadjusted reference-category contrast | 0.7454551789509982 |
| model0_core | race_ethnicity_Other/Multi | race_ethnicity | 0.2427700943790194 | one-hot indicator with all levels retained (drop=None); penalized joint-model coefficient, not an unadjusted reference-category contrast | 1.2747755123699491 |
| model0_core | education_9-11th grade | education | 0.2248619967472025 | one-hot indicator with all levels retained (drop=None); penalized joint-model coefficient, not an unadjusted reference-category contrast | 1.2521499035081085 |
| model0_core | education_<9th grade | education | 0.2543849377257104 | one-hot indicator with all levels retained (drop=None); penalized joint-model coefficient, not an unadjusted reference-category contrast | 1.289668150655285 |
| model0_core | education_College graduate+ | education | -0.2700599330972956 | one-hot indicator with all levels retained (drop=None); penalized joint-model coefficient, not an unadjusted reference-category contrast | 0.7633337440103387 |
| model0_core | education_High school/GED | education | 0.0838787859700336 | one-hot indicator with all levels retained (drop=None); penalized joint-model coefficient, not an unadjusted reference-category contrast | 1.0874970659173562 |
| model0_core | education_Missing | education | -1.5096778511876845 | one-hot indicator with all levels retained (drop=None); penalized joint-model coefficient, not an unadjusted reference-category contrast | 0.2209811553106404 |
| model0_core | education_Some college/AA | education | 0.0162232869736952 | one-hot indicator with all levels retained (drop=None); penalized joint-model coefficient, not an unadjusted reference-category contrast | 1.0163555990376305 |
| model0_core | smoking_Current | smoking | 0.4311845041731063 | one-hot indicator with all levels retained (drop=None); penalized joint-model coefficient, not an unadjusted reference-category contrast | 1.539079490350678 |
| model0_core | smoking_Former | smoking | -0.0806995572655582 | one-hot indicator with all levels retained (drop=None); penalized joint-model coefficient, not an unadjusted reference-category contrast | 0.9224707994644118 |
| model0_core | smoking_Missing | smoking | -1.1389290125508065 | one-hot indicator with all levels retained (drop=None); penalized joint-model coefficient, not an unadjusted reference-category contrast | 0.320161727458646 |
| model0_core | smoking_Never | smoking | -0.4119447112251171 | one-hot indicator with all levels retained (drop=None); penalized joint-model coefficient, not an unadjusted reference-category contrast | 0.6623608961622516 |
| model0_core | diabetes_0.0 | diabetes | -0.9443114851692936 | one-hot indicator with all levels retained (drop=None); penalized joint-model coefficient, not an unadjusted reference-category contrast | 0.3889472746991533 |
| model0_core | diabetes_1.0 | diabetes | -0.2560772916990892 | one-hot indicator with all levels retained (drop=None); penalized joint-model coefficient, not an unadjusted reference-category contrast | 0.7740821363564493 |
| model1_renal | intercept | intercept | -1.23546411303404 | model log-odds intercept after preprocessing | 0.2906998134820755 |
| model1_renal | age | age | 1.1906275802792057 | one training-sample standard deviation after median imputation | 3.2891447621804377 |
| model1_renal | bmi | bmi | 0.1918870994814444 | one training-sample standard deviation after median imputation | 1.2115337264572217 |
| model1_renal | mean_sbp | mean_sbp | -0.0976831995757055 | one training-sample standard deviation after median imputation | 0.9069361760129004 |
| model1_renal | total_cholesterol | total_cholesterol | -0.3183529421705113 | one training-sample standard deviation after median imputation | 0.7273460320199272 |
| model1_renal | hdl_cholesterol | hdl_cholesterol | -0.1030532382649513 | one training-sample standard deviation after median imputation | 0.9020789470851148 |
| model1_renal | egfr | egfr | -0.2468687589699409 | one training-sample standard deviation after median imputation | 0.7812432179681758 |
| model1_renal | log2_uacr | log2_uacr | 0.1913948327252755 | one training-sample standard deviation after median imputation | 1.2109374754490247 |
| model1_renal | sex_Female | sex | -0.6890714527896311 | one-hot indicator with all levels retained (drop=None); penalized joint-model coefficient, not an unadjusted reference-category contrast | 0.5020420224221404 |
| model1_renal | sex_Male | sex | -0.4833121708800765 | one-hot indicator with all levels retained (drop=None); penalized joint-model coefficient, not an unadjusted reference-category contrast | 0.6167372659046204 |
| model1_renal | race_ethnicity_Mexican American | race_ethnicity | -0.4644918292111819 | one-hot indicator with all levels retained (drop=None); penalized joint-model coefficient, not an unadjusted reference-category contrast | 0.6284543862193048 |
| model1_renal | race_ethnicity_Non-Hispanic Asian | race_ethnicity | -0.5296243777673918 | one-hot indicator with all levels retained (drop=None); penalized joint-model coefficient, not an unadjusted reference-category contrast | 0.5888261043201339 |
| model1_renal | race_ethnicity_Non-Hispanic Black | race_ethnicity | -0.0638695818584874 | one-hot indicator with all levels retained (drop=None); penalized joint-model coefficient, not an unadjusted reference-category contrast | 0.9381273403770573 |
| model1_renal | race_ethnicity_Non-Hispanic White | race_ethnicity | -0.110072012896041 | one-hot indicator with all levels retained (drop=None); penalized joint-model coefficient, not an unadjusted reference-category contrast | 0.8957696260088513 |
| model1_renal | race_ethnicity_Other Hispanic | race_ethnicity | -0.249346947763081 | one-hot indicator with all levels retained (drop=None); penalized joint-model coefficient, not an unadjusted reference-category contrast | 0.7793095467714587 |
| model1_renal | race_ethnicity_Other/Multi | race_ethnicity | 0.2450211258264834 | one-hot indicator with all levels retained (drop=None); penalized joint-model coefficient, not an unadjusted reference-category contrast | 1.2776483042961644 |
| model1_renal | education_9-11th grade | education | 0.1929687970211718 | one-hot indicator with all levels retained (drop=None); penalized joint-model coefficient, not an unadjusted reference-category contrast | 1.2128449485534332 |
| model1_renal | education_<9th grade | education | 0.2357376624689567 | one-hot indicator with all levels retained (drop=None); penalized joint-model coefficient, not an unadjusted reference-category contrast | 1.265842188690349 |
| model1_renal | education_College graduate+ | education | -0.2567823028983053 | one-hot indicator with all levels retained (drop=None); penalized joint-model coefficient, not an unadjusted reference-category contrast | 0.7735365921112026 |
| model1_renal | education_High school/GED | education | 0.0744664162794848 | one-hot indicator with all levels retained (drop=None); penalized joint-model coefficient, not an unadjusted reference-category contrast | 1.077309162864884 |
| model1_renal | education_Missing | education | -1.4258840277602578 | one-hot indicator with all levels retained (drop=None); penalized joint-model coefficient, not an unadjusted reference-category contrast | 0.2402959410018947 |
| model1_renal | education_Some college/AA | education | 0.0071098312192413 | one-hot indicator with all levels retained (drop=None); penalized joint-model coefficient, not an unadjusted reference-category contrast | 1.007135166075818 |
| model1_renal | smoking_Current | smoking | 0.3929624550640062 | one-hot indicator with all levels retained (drop=None); penalized joint-model coefficient, not an unadjusted reference-category contrast | 1.481362770614764 |
| model1_renal | smoking_Former | smoking | -0.1163277086978882 | one-hot indicator with all levels retained (drop=None); penalized joint-model coefficient, not an unadjusted reference-category contrast | 0.89018345463172 |
| model1_renal | smoking_Missing | smoking | -1.0030171502354233 | one-hot indicator with all levels retained (drop=None); penalized joint-model coefficient, not an unadjusted reference-category contrast | 0.366771166385407 |
| model1_renal | smoking_Never | smoking | -0.4460012198003932 | one-hot indicator with all levels retained (drop=None); penalized joint-model coefficient, not an unadjusted reference-category contrast | 0.6401829911657065 |
| model1_renal | diabetes_0.0 | diabetes | -0.8751620270398145 | one-hot indicator with all levels retained (drop=None); penalized joint-model coefficient, not an unadjusted reference-category contrast | 0.4167944822310429 |
| model1_renal | diabetes_1.0 | diabetes | -0.2972215966298865 | one-hot indicator with all levels retained (drop=None); penalized joint-model coefficient, not an unadjusted reference-category contrast | 0.7428793745552084 |
| model2_metabolic | intercept | intercept | -1.3032538605332804 | model log-odds intercept after preprocessing | 0.2716464537549997 |
| model2_metabolic | age | age | 1.3149526399310598 | one training-sample standard deviation after median imputation | 3.724574584964968 |
| model2_metabolic | bmi | bmi | 0.0135764749103573 | one training-sample standard deviation after median imputation | 1.013669053736121 |
| model2_metabolic | mean_sbp | mean_sbp | 0.0004944900169492 | one training-sample standard deviation after median imputation | 1.0004946122972924 |
| model2_metabolic | total_cholesterol | total_cholesterol | -0.400498610909816 | one training-sample standard deviation after median imputation | 0.6699859004588553 |
| model2_metabolic | hdl_cholesterol | hdl_cholesterol | -0.0367728054608651 | one training-sample standard deviation after median imputation | 0.9638951021775912 |
| model2_metabolic | tyg_wc | tyg_wc | 0.2428186129019582 | one training-sample standard deviation after median imputation | 1.274837364095353 |
| model2_metabolic | sex_Female | sex | -0.7089562778294524 | one-hot indicator with all levels retained (drop=None); penalized joint-model coefficient, not an unadjusted reference-category contrast | 0.4921576052908365 |
| model2_metabolic | sex_Male | sex | -0.5939753376724736 | one-hot indicator with all levels retained (drop=None); penalized joint-model coefficient, not an unadjusted reference-category contrast | 0.5521280209011095 |
| model2_metabolic | race_ethnicity_Mexican American | race_ethnicity | -0.5532677364079023 | one-hot indicator with all levels retained (drop=None); penalized joint-model coefficient, not an unadjusted reference-category contrast | 0.5750675674912809 |
| model2_metabolic | race_ethnicity_Non-Hispanic Asian | race_ethnicity | -0.6621192638262603 | one-hot indicator with all levels retained (drop=None); penalized joint-model coefficient, not an unadjusted reference-category contrast | 0.5157571499972897 |
| model2_metabolic | race_ethnicity_Non-Hispanic Black | race_ethnicity | 0.0205811837014984 | one-hot indicator with all levels retained (drop=None); penalized joint-model coefficient, not an unadjusted reference-category contrast | 1.0207944367502064 |
| model2_metabolic | race_ethnicity_Non-Hispanic White | race_ethnicity | -0.1515091187396895 | one-hot indicator with all levels retained (drop=None); penalized joint-model coefficient, not an unadjusted reference-category contrast | 0.8594100455007048 |
| model2_metabolic | race_ethnicity_Other Hispanic | race_ethnicity | -0.2371450369627966 | one-hot indicator with all levels retained (drop=None); penalized joint-model coefficient, not an unadjusted reference-category contrast | 0.7888768634156216 |
| model2_metabolic | race_ethnicity_Other/Multi | race_ethnicity | 0.280528356733223 | one-hot indicator with all levels retained (drop=None); penalized joint-model coefficient, not an unadjusted reference-category contrast | 1.3238290815982574 |
| model2_metabolic | education_9-11th grade | education | 0.2675817813566121 | one-hot indicator with all levels retained (drop=None); penalized joint-model coefficient, not an unadjusted reference-category contrast | 1.3068004973836396 |
| model2_metabolic | education_<9th grade | education | 0.1406243156021106 | one-hot indicator with all levels retained (drop=None); penalized joint-model coefficient, not an unadjusted reference-category contrast | 1.150992156954304 |
| model2_metabolic | education_College graduate+ | education | -0.3293167377258433 | one-hot indicator with all levels retained (drop=None); penalized joint-model coefficient, not an unadjusted reference-category contrast | 0.7194151147490174 |
| model2_metabolic | education_High school/GED | education | -0.023204881844488 | one-hot indicator with all levels retained (drop=None); penalized joint-model coefficient, not an unadjusted reference-category contrast | 0.9770622809427156 |
| model2_metabolic | education_Missing | education | -1.3141714097368182 | one-hot indicator with all levels retained (drop=None); penalized joint-model coefficient, not an unadjusted reference-category contrast | 0.2686968706369576 |
| model2_metabolic | education_Some college/AA | education | -0.0444446831534734 | one-hot indicator with all levels retained (drop=None); penalized joint-model coefficient, not an unadjusted reference-category contrast | 0.95652851077101 |
| model2_metabolic | smoking_Current | smoking | 0.7834660850796338 | one-hot indicator with all levels retained (drop=None); penalized joint-model coefficient, not an unadjusted reference-category contrast | 2.189046552948678 |
| model2_metabolic | smoking_Former | smoking | 0.1626587671232369 | one-hot indicator with all levels retained (drop=None); penalized joint-model coefficient, not an unadjusted reference-category contrast | 1.1766351145919984 |
| model2_metabolic | smoking_Missing | smoking | -2.1306647630068154 | one-hot indicator with all levels retained (drop=None); penalized joint-model coefficient, not an unadjusted reference-category contrast | 0.1187583214674947 |
| model2_metabolic | smoking_Never | smoking | -0.1183917046979428 | one-hot indicator with all levels retained (drop=None); penalized joint-model coefficient, not an unadjusted reference-category contrast | 0.8883480143643239 |
| model2_metabolic | diabetes_0.0 | diabetes | -0.9159786047316858 | one-hot indicator with all levels retained (drop=None); penalized joint-model coefficient, not an unadjusted reference-category contrast | 0.4001248703436857 |
| model2_metabolic | diabetes_1.0 | diabetes | -0.3869530107702223 | one-hot indicator with all levels retained (drop=None); penalized joint-model coefficient, not an unadjusted reference-category contrast | 0.6791230056438604 |
| model3_inflammatory | intercept | intercept | -1.2369483026720105 | model log-odds intercept after preprocessing | 0.2902686798523208 |
| model3_inflammatory | age | age | 1.3764156727314194 | one training-sample standard deviation after median imputation | 3.960679782301305 |
| model3_inflammatory | bmi | bmi | 0.188671770161095 | one training-sample standard deviation after median imputation | 1.2076445024626 |
| model3_inflammatory | mean_sbp | mean_sbp | -0.0566704529547794 | one training-sample standard deviation after median imputation | 0.9449054088487016 |
| model3_inflammatory | total_cholesterol | total_cholesterol | -0.3152529142228035 | one training-sample standard deviation after median imputation | 0.7296043236218358 |
| model3_inflammatory | hdl_cholesterol | hdl_cholesterol | -0.1210813762784309 | one training-sample standard deviation after median imputation | 0.8859618603803031 |
| model3_inflammatory | siri | siri | 0.1048771077372862 | one training-sample standard deviation after median imputation | 1.1105741210024929 |
| model3_inflammatory | sex_Female | sex | -0.687886496085722 | one-hot indicator with all levels retained (drop=None); penalized joint-model coefficient, not an unadjusted reference-category contrast | 0.5026372730857352 |
| model3_inflammatory | sex_Male | sex | -0.5488379107546403 | one-hot indicator with all levels retained (drop=None); penalized joint-model coefficient, not an unadjusted reference-category contrast | 0.5776206672725352 |
| model3_inflammatory | race_ethnicity_Mexican American | race_ethnicity | -0.5158239450902026 | one-hot indicator with all levels retained (drop=None); penalized joint-model coefficient, not an unadjusted reference-category contrast | 0.5970084896984652 |
| model3_inflammatory | race_ethnicity_Non-Hispanic Asian | race_ethnicity | -0.6115068431075719 | one-hot indicator with all levels retained (drop=None); penalized joint-model coefficient, not an unadjusted reference-category contrast | 0.5425327411124838 |
| model3_inflammatory | race_ethnicity_Non-Hispanic Black | race_ethnicity | 0.0583616242166664 | one-hot indicator with all levels retained (drop=None); penalized joint-model coefficient, not an unadjusted reference-category contrast | 1.0600982836144182 |
| model3_inflammatory | race_ethnicity_Non-Hispanic White | race_ethnicity | -0.1405687893773304 | one-hot indicator with all levels retained (drop=None); penalized joint-model coefficient, not an unadjusted reference-category contrast | 0.868863894270735 |
| model3_inflammatory | race_ethnicity_Other Hispanic | race_ethnicity | -0.2807433734772447 | one-hot indicator with all levels retained (drop=None); penalized joint-model coefficient, not an unadjusted reference-category contrast | 0.7552221206406664 |
| model3_inflammatory | race_ethnicity_Other/Multi | race_ethnicity | 0.253556919995371 | one-hot indicator with all levels retained (drop=None); penalized joint-model coefficient, not an unadjusted reference-category contrast | 1.2886007245451476 |
| model3_inflammatory | education_9-11th grade | education | 0.2336978888800303 | one-hot indicator with all levels retained (drop=None); penalized joint-model coefficient, not an unadjusted reference-category contrast | 1.2632627888163162 |
| model3_inflammatory | education_<9th grade | education | 0.2846712028856569 | one-hot indicator with all levels retained (drop=None); penalized joint-model coefficient, not an unadjusted reference-category contrast | 1.3293248780746396 |
| model3_inflammatory | education_College graduate+ | education | -0.2459085525136458 | one-hot indicator with all levels retained (drop=None); penalized joint-model coefficient, not an unadjusted reference-category contrast | 0.7819937330170389 |
| model3_inflammatory | education_High school/GED | education | 0.0892138316063887 | one-hot indicator with all levels retained (drop=None); penalized joint-model coefficient, not an unadjusted reference-category contrast | 1.093314416510752 |
| model3_inflammatory | education_Missing | education | -1.6256465129897022 | one-hot indicator with all levels retained (drop=None); penalized joint-model coefficient, not an unadjusted reference-category contrast | 0.1967844103919604 |
| model3_inflammatory | education_Some college/AA | education | 0.0272477352909377 | one-hot indicator with all levels retained (drop=None); penalized joint-model coefficient, not an unadjusted reference-category contrast | 1.0276223495538168 |
| model3_inflammatory | smoking_Current | smoking | 0.4495850313491129 | one-hot indicator with all levels retained (drop=None); penalized joint-model coefficient, not an unadjusted reference-category contrast | 1.5676615201105906 |
| model3_inflammatory | smoking_Former | smoking | -0.0459379053361578 | one-hot indicator with all levels retained (drop=None); penalized joint-model coefficient, not an unadjusted reference-category contrast | 0.9551012670415504 |
| model3_inflammatory | smoking_Missing | smoking | -1.2764271961830247 | one-hot indicator with all levels retained (drop=None); penalized joint-model coefficient, not an unadjusted reference-category contrast | 0.2790324498597299 |
| model3_inflammatory | smoking_Never | smoking | -0.3639443366702228 | one-hot indicator with all levels retained (drop=None); penalized joint-model coefficient, not an unadjusted reference-category contrast | 0.6949298757771905 |
| model3_inflammatory | diabetes_0.0 | diabetes | -0.9516415477696132 | one-hot indicator with all levels retained (drop=None); penalized joint-model coefficient, not an unadjusted reference-category contrast | 0.3861066903764999 |
| model3_inflammatory | diabetes_1.0 | diabetes | -0.2850828590707271 | one-hot indicator with all levels retained (drop=None); penalized joint-model coefficient, not an unadjusted reference-category contrast | 0.751951945698549 |
| model4_combined | intercept | intercept | -0.9941369471502908 | model log-odds intercept after preprocessing | 0.370042673152242 |
| model4_combined | age | age | 1.0363985975472392 | one training-sample standard deviation after median imputation | 2.8190461908640554 |
| model4_combined | bmi | bmi | 0.0249332255890613 | one training-sample standard deviation after median imputation | 1.0252466579970272 |
| model4_combined | mean_sbp | mean_sbp | -0.0524428415747163 | one training-sample standard deviation after median imputation | 0.94890855762464 |
| model4_combined | total_cholesterol | total_cholesterol | -0.3812277719307365 | one training-sample standard deviation after median imputation | 0.6830222985932515 |
| model4_combined | hdl_cholesterol | hdl_cholesterol | -0.0240594736220649 | one training-sample standard deviation after median imputation | 0.9762276482371612 |
| model4_combined | egfr | egfr | -0.3100707836356492 | one training-sample standard deviation after median imputation | 0.7333950420195317 |
| model4_combined | log2_uacr | log2_uacr | 0.1961188817520568 | one training-sample standard deviation after median imputation | 1.216671536781096 |
| model4_combined | tyg_wc | tyg_wc | 0.2187224368343602 | one training-sample standard deviation after median imputation | 1.2444858053249668 |
| model4_combined | siri | siri | 0.0634266755974529 | one training-sample standard deviation after median imputation | 1.0654813571531954 |
| model4_combined | sex_Female | sex | -0.5682365986291854 | one-hot indicator with all levels retained (drop=None); penalized joint-model coefficient, not an unadjusted reference-category contrast | 0.5665235668249757 |
| model4_combined | sex_Male | sex | -0.4168887909710728 | one-hot indicator with all levels retained (drop=None); penalized joint-model coefficient, not an unadjusted reference-category contrast | 0.6590942130960249 |
| model4_combined | race_ethnicity_Mexican American | race_ethnicity | -0.4144590700724284 | one-hot indicator with all levels retained (drop=None); penalized joint-model coefficient, not an unadjusted reference-category contrast | 0.6606975751521148 |
| model4_combined | race_ethnicity_Non-Hispanic Asian | race_ethnicity | -0.6544159246835892 | one-hot indicator with all levels retained (drop=None); penalized joint-model coefficient, not an unadjusted reference-category contrast | 0.5197455444936284 |
| model4_combined | race_ethnicity_Non-Hispanic Black | race_ethnicity | -0.0234110515352902 | one-hot indicator with all levels retained (drop=None); penalized joint-model coefficient, not an unadjusted reference-category contrast | 0.976860861078408 |
| model4_combined | race_ethnicity_Non-Hispanic White | race_ethnicity | -0.0889916475921594 | one-hot indicator with all levels retained (drop=None); penalized joint-model coefficient, not an unadjusted reference-category contrast | 0.9148532147697048 |
| model4_combined | race_ethnicity_Other Hispanic | race_ethnicity | -0.1368680985581361 | one-hot indicator with all levels retained (drop=None); penalized joint-model coefficient, not an unadjusted reference-category contrast | 0.872085247847797 |
| model4_combined | race_ethnicity_Other/Multi | race_ethnicity | 0.3330204028413422 | one-hot indicator with all levels retained (drop=None); penalized joint-model coefficient, not an unadjusted reference-category contrast | 1.3951757637291684 |
| model4_combined | education_9-11th grade | education | 0.09311182660219 | one-hot indicator with all levels retained (drop=None); penalized joint-model coefficient, not an unadjusted reference-category contrast | 1.0975844675472095 |
| model4_combined | education_<9th grade | education | -0.035616239458267 | one-hot indicator with all levels retained (drop=None); penalized joint-model coefficient, not an unadjusted reference-category contrast | 0.9650105554061416 |
| model4_combined | education_College graduate+ | education | -0.478118695834232 | one-hot indicator with all levels retained (drop=None); penalized joint-model coefficient, not an unadjusted reference-category contrast | 0.6199486072975616 |
| model4_combined | education_High school/GED | education | -0.2092360588099339 | one-hot indicator with all levels retained (drop=None); penalized joint-model coefficient, not an unadjusted reference-category contrast | 0.811203721254918 |
| model4_combined | education_Missing | education | -0.1385090326994443 | one-hot indicator with all levels retained (drop=None); penalized joint-model coefficient, not an unadjusted reference-category contrast | 0.8706553868651689 |
| model4_combined | education_Some college/AA | education | -0.2167571894005699 | one-hot indicator with all levels retained (drop=None); penalized joint-model coefficient, not an unadjusted reference-category contrast | 0.8051254385633911 |
| model4_combined | smoking_Current | smoking | 0.2333544537843174 | one-hot indicator with all levels retained (drop=None); penalized joint-model coefficient, not an unadjusted reference-category contrast | 1.2628290145304486 |
| model4_combined | smoking_Former | smoking | -0.3603539418866615 | one-hot indicator with all levels retained (drop=None); penalized joint-model coefficient, not an unadjusted reference-category contrast | 0.6974294328913988 |
| model4_combined | smoking_Missing | smoking | -0.1926413154732986 | one-hot indicator with all levels retained (drop=None); penalized joint-model coefficient, not an unadjusted reference-category contrast | 0.8247777561088128 |
| model4_combined | smoking_Never | smoking | -0.6654845860246168 | one-hot indicator with all levels retained (drop=None); penalized joint-model coefficient, not an unadjusted reference-category contrast | 0.5140243783143073 |
| model4_combined | diabetes_0.0 | diabetes | -0.7120258371741367 | one-hot indicator with all levels retained (drop=None); penalized joint-model coefficient, not an unadjusted reference-category contrast | 0.4906492145462736 |
| model4_combined | diabetes_1.0 | diabetes | -0.2730995524261207 | one-hot indicator with all levels retained (drop=None); penalized joint-model coefficient, not an unadjusted reference-category contrast | 0.7610170227667733 |
| model0_paired_metabolic | intercept | intercept | -1.336976356349782 | model log-odds intercept after preprocessing | 0.2626385947324231 |
| model0_paired_metabolic | age | age | 1.3434894808576012 | one training-sample standard deviation after median imputation | 3.83239326288824 |
| model0_paired_metabolic | bmi | bmi | 0.1809932437976344 | one training-sample standard deviation after median imputation | 1.198407082585082 |
| model0_paired_metabolic | mean_sbp | mean_sbp | 0.0073690115939613 | one training-sample standard deviation after median imputation | 1.0073962295753618 |
| model0_paired_metabolic | total_cholesterol | total_cholesterol | -0.3612814474697692 | one training-sample standard deviation after median imputation | 0.6967828630934689 |
| model0_paired_metabolic | hdl_cholesterol | hdl_cholesterol | -0.0995363063772081 | one training-sample standard deviation after median imputation | 0.9052570826667532 |
| model0_paired_metabolic | sex_Female | sex | -0.750418841730982 | one-hot indicator with all levels retained (drop=None); penalized joint-model coefficient, not an unadjusted reference-category contrast | 0.4721687473438758 |
| model0_paired_metabolic | sex_Male | sex | -0.5765207111687296 | one-hot indicator with all levels retained (drop=None); penalized joint-model coefficient, not an unadjusted reference-category contrast | 0.5618498075436509 |
| model0_paired_metabolic | race_ethnicity_Mexican American | race_ethnicity | -0.557759148172476 | one-hot indicator with all levels retained (drop=None); penalized joint-model coefficient, not an unadjusted reference-category contrast | 0.5724904939346975 |
| model0_paired_metabolic | race_ethnicity_Non-Hispanic Asian | race_ethnicity | -0.731860876359077 | one-hot indicator with all levels retained (drop=None); penalized joint-model coefficient, not an unadjusted reference-category contrast | 0.481013050917872 |
| model0_paired_metabolic | race_ethnicity_Non-Hispanic Black | race_ethnicity | 0.000267683662886 | one-hot indicator with all levels retained (drop=None); penalized joint-model coefficient, not an unadjusted reference-category contrast | 1.0002677194933547 |
| model0_paired_metabolic | race_ethnicity_Non-Hispanic White | race_ethnicity | -0.1067084789394807 | one-hot indicator with all levels retained (drop=None); penalized joint-model coefficient, not an unadjusted reference-category contrast | 0.8987876503315003 |
| model0_paired_metabolic | race_ethnicity_Other Hispanic | race_ethnicity | -0.2306093421808012 | one-hot indicator with all levels retained (drop=None); penalized joint-model coefficient, not an unadjusted reference-category contrast | 0.7940496071396355 |
| model0_paired_metabolic | race_ethnicity_Other/Multi | race_ethnicity | 0.2997306090892556 | one-hot indicator with all levels retained (drop=None); penalized joint-model coefficient, not an unadjusted reference-category contrast | 1.34949521685867 |
| model0_paired_metabolic | education_9-11th grade | education | 0.2952665638961887 | one-hot indicator with all levels retained (drop=None); penalized joint-model coefficient, not an unadjusted reference-category contrast | 1.3434844354045643 |
| model0_paired_metabolic | education_<9th grade | education | 0.1727513136694013 | one-hot indicator with all levels retained (drop=None); penalized joint-model coefficient, not an unadjusted reference-category contrast | 1.1885704870943858 |
| model0_paired_metabolic | education_College graduate+ | education | -0.3051921216464295 | one-hot indicator with all levels retained (drop=None); penalized joint-model coefficient, not an unadjusted reference-category contrast | 0.7369817706383266 |
| model0_paired_metabolic | education_High school/GED | education | 0.0009225478621203 | one-hot indicator with all levels retained (drop=None); penalized joint-model coefficient, not an unadjusted reference-category contrast | 1.000922973540292 |
| model0_paired_metabolic | education_Missing | education | -1.479486469244032 | one-hot indicator with all levels retained (drop=None); penalized joint-model coefficient, not an unadjusted reference-category contrast | 0.2277546173587581 |
| model0_paired_metabolic | education_Some college/AA | education | -0.0112013874369641 | one-hot indicator with all levels retained (drop=None); penalized joint-model coefficient, not an unadjusted reference-category contrast | 0.988861114516086 |
| model0_paired_metabolic | smoking_Current | smoking | 0.8492710153422457 | one-hot indicator with all levels retained (drop=None); penalized joint-model coefficient, not an unadjusted reference-category contrast | 2.337941906781303 |
| model0_paired_metabolic | smoking_Former | smoking | 0.2257465787321285 | one-hot indicator with all levels retained (drop=None); penalized joint-model coefficient, not an unadjusted reference-category contrast | 1.2532580227940997 |
| model0_paired_metabolic | smoking_Missing | smoking | -2.321797342781983 | one-hot indicator with all levels retained (drop=None); penalized joint-model coefficient, not an unadjusted reference-category contrast | 0.0980971129230729 |
| model0_paired_metabolic | smoking_Never | smoking | -0.0801598041920998 | one-hot indicator with all levels retained (drop=None); penalized joint-model coefficient, not an unadjusted reference-category contrast | 0.922968840311046 |
| model0_paired_metabolic | diabetes_0.0 | diabetes | -0.977243841487493 | one-hot indicator with all levels retained (drop=None); penalized joint-model coefficient, not an unadjusted reference-category contrast | 0.376346942550327 |
| model0_paired_metabolic | diabetes_1.0 | diabetes | -0.3496957114122149 | one-hot indicator with all levels retained (drop=None); penalized joint-model coefficient, not an unadjusted reference-category contrast | 0.7049025508897515 |
| model0_paired_combined | intercept | intercept | -1.336976356349782 | model log-odds intercept after preprocessing | 0.2626385947324231 |
| model0_paired_combined | age | age | 1.3434894808576012 | one training-sample standard deviation after median imputation | 3.83239326288824 |
| model0_paired_combined | bmi | bmi | 0.1809932437976344 | one training-sample standard deviation after median imputation | 1.198407082585082 |
| model0_paired_combined | mean_sbp | mean_sbp | 0.0073690115939613 | one training-sample standard deviation after median imputation | 1.0073962295753618 |
| model0_paired_combined | total_cholesterol | total_cholesterol | -0.3612814474697692 | one training-sample standard deviation after median imputation | 0.6967828630934689 |
| model0_paired_combined | hdl_cholesterol | hdl_cholesterol | -0.0995363063772081 | one training-sample standard deviation after median imputation | 0.9052570826667532 |
| model0_paired_combined | sex_Female | sex | -0.750418841730982 | one-hot indicator with all levels retained (drop=None); penalized joint-model coefficient, not an unadjusted reference-category contrast | 0.4721687473438758 |
| model0_paired_combined | sex_Male | sex | -0.5765207111687296 | one-hot indicator with all levels retained (drop=None); penalized joint-model coefficient, not an unadjusted reference-category contrast | 0.5618498075436509 |
| model0_paired_combined | race_ethnicity_Mexican American | race_ethnicity | -0.557759148172476 | one-hot indicator with all levels retained (drop=None); penalized joint-model coefficient, not an unadjusted reference-category contrast | 0.5724904939346975 |
| model0_paired_combined | race_ethnicity_Non-Hispanic Asian | race_ethnicity | -0.731860876359077 | one-hot indicator with all levels retained (drop=None); penalized joint-model coefficient, not an unadjusted reference-category contrast | 0.481013050917872 |
| model0_paired_combined | race_ethnicity_Non-Hispanic Black | race_ethnicity | 0.000267683662886 | one-hot indicator with all levels retained (drop=None); penalized joint-model coefficient, not an unadjusted reference-category contrast | 1.0002677194933547 |
| model0_paired_combined | race_ethnicity_Non-Hispanic White | race_ethnicity | -0.1067084789394807 | one-hot indicator with all levels retained (drop=None); penalized joint-model coefficient, not an unadjusted reference-category contrast | 0.8987876503315003 |
| model0_paired_combined | race_ethnicity_Other Hispanic | race_ethnicity | -0.2306093421808012 | one-hot indicator with all levels retained (drop=None); penalized joint-model coefficient, not an unadjusted reference-category contrast | 0.7940496071396355 |
| model0_paired_combined | race_ethnicity_Other/Multi | race_ethnicity | 0.2997306090892556 | one-hot indicator with all levels retained (drop=None); penalized joint-model coefficient, not an unadjusted reference-category contrast | 1.34949521685867 |
| model0_paired_combined | education_9-11th grade | education | 0.2952665638961887 | one-hot indicator with all levels retained (drop=None); penalized joint-model coefficient, not an unadjusted reference-category contrast | 1.3434844354045643 |
| model0_paired_combined | education_<9th grade | education | 0.1727513136694013 | one-hot indicator with all levels retained (drop=None); penalized joint-model coefficient, not an unadjusted reference-category contrast | 1.1885704870943858 |
| model0_paired_combined | education_College graduate+ | education | -0.3051921216464295 | one-hot indicator with all levels retained (drop=None); penalized joint-model coefficient, not an unadjusted reference-category contrast | 0.7369817706383266 |
| model0_paired_combined | education_High school/GED | education | 0.0009225478621203 | one-hot indicator with all levels retained (drop=None); penalized joint-model coefficient, not an unadjusted reference-category contrast | 1.000922973540292 |
| model0_paired_combined | education_Missing | education | -1.479486469244032 | one-hot indicator with all levels retained (drop=None); penalized joint-model coefficient, not an unadjusted reference-category contrast | 0.2277546173587581 |
| model0_paired_combined | education_Some college/AA | education | -0.0112013874369641 | one-hot indicator with all levels retained (drop=None); penalized joint-model coefficient, not an unadjusted reference-category contrast | 0.988861114516086 |
| model0_paired_combined | smoking_Current | smoking | 0.8492710153422457 | one-hot indicator with all levels retained (drop=None); penalized joint-model coefficient, not an unadjusted reference-category contrast | 2.337941906781303 |
| model0_paired_combined | smoking_Former | smoking | 0.2257465787321285 | one-hot indicator with all levels retained (drop=None); penalized joint-model coefficient, not an unadjusted reference-category contrast | 1.2532580227940997 |
| model0_paired_combined | smoking_Missing | smoking | -2.321797342781983 | one-hot indicator with all levels retained (drop=None); penalized joint-model coefficient, not an unadjusted reference-category contrast | 0.0980971129230729 |
| model0_paired_combined | smoking_Never | smoking | -0.0801598041920998 | one-hot indicator with all levels retained (drop=None); penalized joint-model coefficient, not an unadjusted reference-category contrast | 0.922968840311046 |
| model0_paired_combined | diabetes_0.0 | diabetes | -0.977243841487493 | one-hot indicator with all levels retained (drop=None); penalized joint-model coefficient, not an unadjusted reference-category contrast | 0.376346942550327 |
| model0_paired_combined | diabetes_1.0 | diabetes | -0.3496957114122149 | one-hot indicator with all levels retained (drop=None); penalized joint-model coefficient, not an unadjusted reference-category contrast | 0.7049025508897515 |

Note. Categorical terms use one-hot encoding with all observed levels retained (drop=None), including the explicit Missing level. They are penalized joint-model coefficients and are not conventional contrasts against an omitted reference category.

Abbreviations. AUROC, area under the receiver operating characteristic curve; CI, confidence interval; CVD, cardiovascular disease; DCA, decision-curve analysis; eGFR, estimated glomerular filtration rate; I2, percentage of total variability attributed to between-cycle heterogeneity; MEC, mobile examination center; MI, multiple imputation; n, number; PR-AUC, area under the precision-recall curve; SIRI, systemic inflammation response index; TyG-WC, triglyceride-glucose waist circumference index; UACR, urine albumin-to-creatinine ratio. GED, General Educational Development; AA, associate of arts.
