# Supplementary material for: Incremental value of renal, metabolic, and inflammatory markers for prevalent self-reported cardiovascular disease across NHANES cycles: A repeated cross-sectional validation study
Source: Medicine (Baltimore). 2026 Nov 6;105(45):e51425. doi: 10.1097/MD.0000000000051425 (PMC13747496; doi:10.1097/MD.0000000000051425)
Supplement: Supplementary file 3 [file medi-105-e51425-s003.docx]

**Supplemental Table S10**

Incremental value of renal, metabolic, and inflammatory markers for prevalent self-reported cardiovascular disease across NHANES cycles: a repeated cross-sectional validation study

First author: Haoyang Li; second author: Xiang Li

# Table S10. Selected hyperparameters and hashes for final frozen models

| **model** | **algorithm** | **training_domain** | **n_training** | **events_training** | **weight_variable** | **selected_candidate_id** | **mean_inner_weighted_log_loss** | **selected_hyperparameters** | **model_sha256** |
| --- | --- | --- | --- | --- | --- | --- | --- | --- | --- |
| model0_core | elastic_net | MEC examination sample | 37482 | 4222 | combined_mec_weight | elastic_net_0001 | 0.226423770605174 | {"C": 0.003, "class_weight": null, "l1_ratio": 0.0, "max_iter": 5000, "solver": "saga", "tol": 0.0001} | 2d78e7691f32ea2b4f151cd870c88643a87a7ad646587540ac2dc5983a729e2c |
| model1_renal | elastic_net | MEC examination sample | 37482 | 4222 | combined_mec_weight | elastic_net_0000 | 0.223671758915816 | {"C": 0.001, "class_weight": null, "l1_ratio": 0.0, "max_iter": 5000, "solver": "saga", "tol": 0.0001} | 19b52b6d3b9418e2862a289de908540601cb0c0cafbdd277f9b72d01f1e1b6c4 |
| model2_metabolic | elastic_net | fasting subsample | 15956 | 1813 | combined_fasting_weight | elastic_net_0005 | 0.2347825846218264 | {"C": 0.3, "class_weight": null, "l1_ratio": 0.0, "max_iter": 5000, "solver": "saga", "tol": 0.0001} | 905b92bc4074e8c264917dee084dcb6309b168a4f4f58e83c09560df2e7a2c77 |
| model3_inflammatory | elastic_net | MEC examination sample | 37482 | 4222 | combined_mec_weight | elastic_net_0005 | 0.2259010100354521 | {"C": 0.3, "class_weight": null, "l1_ratio": 0.0, "max_iter": 5000, "solver": "saga", "tol": 0.0001} | cc8418be665c49b6642617317d81f42468379652b20fe7135d066d8fb11aa4bc |
| model4_combined | elastic_net | fasting subsample | 15956 | 1813 | combined_fasting_weight | elastic_net_0000 | 0.2309287612357134 | {"C": 0.001, "class_weight": null, "l1_ratio": 0.0, "max_iter": 5000, "solver": "saga", "tol": 0.0001} | 9c0c2295e2aadb40964bdf12d8e4dee6a7408f2a78296a514ba8dfa3b52f3230 |
| model0_paired_metabolic | elastic_net | fasting subsample | 15956 | 1813 | combined_fasting_weight | elastic_net_0002 | 0.2352891506735363 | {"C": 0.01, "class_weight": null, "l1_ratio": 0.0, "max_iter": 5000, "solver": "saga", "tol": 0.0001} | 953352a0052dd3b69e4013c948de0506eccee0fddd72495d088d5875ce4f28f0 |
| model0_paired_combined | elastic_net | fasting subsample | 15956 | 1813 | combined_fasting_weight | elastic_net_0002 | 0.2352891506735363 | {"C": 0.01, "class_weight": null, "l1_ratio": 0.0, "max_iter": 5000, "solver": "saga", "tol": 0.0001} | 953352a0052dd3b69e4013c948de0506eccee0fddd72495d088d5875ce4f28f0 |
| model0_core | xgboost | MEC examination sample | 37482 | 4222 | combined_mec_weight | xgboost_0196 | 0.2242406174655892 | {"colsample_bytree": 0.8, "learning_rate": 0.03, "max_depth": 3, "min_child_weight": 5, "n_estimators": 500, "reg_alpha": 0.1, "reg_lambda": 1, "scale_pos_weight": 1, "subsample": 0.8} | 9f538da99aedc06121e2df7bb12259b534f8e973d1b378a91dfe158b689eae43 |
| model1_renal | xgboost | MEC examination sample | 37482 | 4222 | combined_mec_weight | xgboost_0032 | 0.2220819933712003 | {"colsample_bytree": 0.8, "learning_rate": 0.1, "max_depth": 2, "min_child_weight": 5, "n_estimators": 200, "reg_alpha": 0.1, "reg_lambda": 5, "scale_pos_weight": 1, "subsample": 0.8} | 3632013712a5f4cb740802517e24f7f57cfd9de7a026ad69676d3d5000508685 |
| model2_metabolic | xgboost | fasting subsample | 15956 | 1813 | combined_fasting_weight | xgboost_0133 | 0.2342771541111401 | {"colsample_bytree": 1.0, "learning_rate": 0.03, "max_depth": 3, "min_child_weight": 5, "n_estimators": 200, "reg_alpha": 0.1, "reg_lambda": 1, "scale_pos_weight": 1, "subsample": 0.8} | 6ab55703d69698d2d3a9a680eb9a889c8a8d9eb1fc5b9343859d93fe4e48b754 |
| model3_inflammatory | xgboost | MEC examination sample | 37482 | 4222 | combined_mec_weight | xgboost_0069 | 0.2240263223448768 | {"colsample_bytree": 1.0, "learning_rate": 0.03, "max_depth": 2, "min_child_weight": 5, "n_estimators": 500, "reg_alpha": 0.1, "reg_lambda": 1, "scale_pos_weight": 1, "subsample": 0.8} | 51e3cb9a96dd05f2ee2d35f87f82f3bacd3cf3edfc5971bec63fe9c3536755d1 |
| model4_combined | xgboost | fasting subsample | 15956 | 1813 | combined_fasting_weight | xgboost_0068 | 0.2315839388610888 | {"colsample_bytree": 0.8, "learning_rate": 0.03, "max_depth": 2, "min_child_weight": 5, "n_estimators": 500, "reg_alpha": 0.1, "reg_lambda": 1, "scale_pos_weight": 1, "subsample": 0.8} | 6659efbbef2a4a53ee7ec12a44b8bf37f8aa8921200c0a9ee251507e7f5fd6d5 |
| model0_paired_metabolic | xgboost | fasting subsample | 15956 | 1813 | combined_fasting_weight | xgboost_0128 | 0.233882753168486 | {"colsample_bytree": 0.8, "learning_rate": 0.03, "max_depth": 3, "min_child_weight": 5, "n_estimators": 200, "reg_alpha": 0.1, "reg_lambda": 5, "scale_pos_weight": 1, "subsample": 0.8} | 62e15e7b6251504035ff1d8efa970b75c09d61f1d8bafe4678d42a603ef12ae2 |
| model0_paired_combined | xgboost | fasting subsample | 15956 | 1813 | combined_fasting_weight | xgboost_0128 | 0.233882753168486 | {"colsample_bytree": 0.8, "learning_rate": 0.03, "max_depth": 3, "min_child_weight": 5, "n_estimators": 200, "reg_alpha": 0.1, "reg_lambda": 5, "scale_pos_weight": 1, "subsample": 0.8} | 62e15e7b6251504035ff1d8efa970b75c09d61f1d8bafe4678d42a603ef12ae2 |

Abbreviations. AUROC, area under the receiver operating characteristic curve; CI, confidence interval; CVD, cardiovascular disease; DCA, decision-curve analysis; eGFR, estimated glomerular filtration rate; I2, percentage of total variability attributed to between-cycle heterogeneity; MEC, mobile examination center; MI, multiple imputation; n, number; PR-AUC, area under the precision-recall curve; SIRI, systemic inflammation response index; TyG-WC, triglyceride-glucose waist circumference index; UACR, urine albumin-to-creatinine ratio.
