# Supplementary material for: Incremental value of renal, metabolic, and inflammatory markers for prevalent self-reported cardiovascular disease across NHANES cycles: A repeated cross-sectional validation study
Source: Medicine (Baltimore). 2026 Nov 6;105(45):e51425. doi: 10.1097/MD.0000000000051425 (PMC13747496; doi:10.1097/MD.0000000000051425)
Supplement: Supplementary file 4 [file medi-105-e51425-s004.docx]

**Supplemental Table S4**

Incremental value of renal, metabolic, and inflammatory markers for prevalent self-reported cardiovascular disease across NHANES cycles: a repeated cross-sectional validation study

First author: Haoyang Li; second author: Xiang Li

# Table S4. Inventory of the frozen development models

| **Model** | **Algorithm** | **Training period** | **Temporal validation** | **Frozen model SHA-256** |
| --- | --- | --- | --- | --- |
| Model 0 (core clinical) | Elastic Net | NHANES 2005-2018 | locked | 2d78e7691f32ea2b4f151cd870c88643a87a7ad646587540ac2dc5983a729e2c |
| Model 0 (core clinical) | XGBoost | NHANES 2005-2018 | locked | 9f538da99aedc06121e2df7bb12259b534f8e973d1b378a91dfe158b689eae43 |
| Model 1 (+ renal) | Elastic Net | NHANES 2005-2018 | locked | 19b52b6d3b9418e2862a289de908540601cb0c0cafbdd277f9b72d01f1e1b6c4 |
| Model 1 (+ renal) | XGBoost | NHANES 2005-2018 | locked | 3632013712a5f4cb740802517e24f7f57cfd9de7a026ad69676d3d5000508685 |
| Model 2 (+ TyG-WC) | Elastic Net | NHANES 2005-2018 | locked | 905b92bc4074e8c264917dee084dcb6309b168a4f4f58e83c09560df2e7a2c77 |
| Model 2 (+ TyG-WC) | XGBoost | NHANES 2005-2018 | locked | 6ab55703d69698d2d3a9a680eb9a889c8a8d9eb1fc5b9343859d93fe4e48b754 |
| Model 3 (+ SIRI) | Elastic Net | NHANES 2005-2018 | locked | cc8418be665c49b6642617317d81f42468379652b20fe7135d066d8fb11aa4bc |
| Model 3 (+ SIRI) | XGBoost | NHANES 2005-2018 | locked | 51e3cb9a96dd05f2ee2d35f87f82f3bacd3cf3edfc5971bec63fe9c3536755d1 |
| Model 4 (combined) | Elastic Net | NHANES 2005-2018 | locked | 9c0c2295e2aadb40964bdf12d8e4dee6a7408f2a78296a514ba8dfa3b52f3230 |
| Model 4 (combined) | XGBoost | NHANES 2005-2018 | locked | 6659efbbef2a4a53ee7ec12a44b8bf37f8aa8921200c0a9ee251507e7f5fd6d5 |

Abbreviations. AUROC, area under the receiver operating characteristic curve; CI, confidence interval; CVD, cardiovascular disease; DCA, decision-curve analysis; eGFR, estimated glomerular filtration rate; I2, percentage of total variability attributed to between-cycle heterogeneity; MEC, mobile examination center; MI, multiple imputation; n, number; PR-AUC, area under the precision-recall curve; SIRI, systemic inflammation response index; TyG-WC, triglyceride-glucose waist circumference index; UACR, urine albumin-to-creatinine ratio. NHANES, National Health and Nutrition Examination Survey; SHA-256, 256-bit Secure Hash Algorithm.
