# Supplementary material for: Incremental value of renal, metabolic, and inflammatory markers for prevalent self-reported cardiovascular disease across NHANES cycles: A repeated cross-sectional validation study
Source: Medicine (Baltimore). 2026 Nov 6;105(45):e51425. doi: 10.1097/MD.0000000000051425 (PMC13747496; doi:10.1097/MD.0000000000051425)
Supplement: Supplementary file 5 [file medi-105-e51425-s006.docx]

**Supplemental Table S5**

Incremental value of renal, metabolic, and inflammatory markers for prevalent self-reported cardiovascular disease across NHANES cycles: a repeated cross-sectional validation study

First author: Haoyang Li; second author: Xiang Li

# Table S5. Analytic sample counts by survey cycle and analysis domain

| **NHANES cycle** | **Outcome-available n** | **CVD events** | **Survey-weighted prevalence, %** | **Primary component-weight analysis n (events)** | **Fasting analysis n (events)** |
| --- | --- | --- | --- | --- | --- |
| 2005-2006 | 4643 | 559 | 9.0 | 4448 (533) | 1836 (216) |
| 2007-2008 | 5877 | 716 | 8.6 | 5649 (677) | 2337 (288) |
| 2009-2010 | 6148 | 667 | 8.1 | 5990 (646) | 2594 (273) |
| 2011-2012 | 5500 | 581 | 8.7 | 5260 (550) | 2294 (239) |
| 2013-2014 | 5703 | 594 | 8.8 | 5522 (573) | 2370 (244) |
| 2015-2016 | 5649 | 634 | 8.7 | 5404 (597) | 2246 (266) |
| 2017-2018 | 5513 | 690 | 9.6 | 5209 (646) | 2279 (287) |
| 2021-2023 | 7766 | 982 | 9.7 | 5755 (739) | 2940 (355) |

Abbreviations. AUROC, area under the receiver operating characteristic curve; CI, confidence interval; CVD, cardiovascular disease; DCA, decision-curve analysis; eGFR, estimated glomerular filtration rate; I2, percentage of total variability attributed to between-cycle heterogeneity; MEC, mobile examination center; MI, multiple imputation; n, number; PR-AUC, area under the precision-recall curve; SIRI, systemic inflammation response index; TyG-WC, triglyceride-glucose waist circumference index; UACR, urine albumin-to-creatinine ratio. NHANES, National Health and Nutrition Examination Survey.
