# Supplementary material for: Incremental value of renal, metabolic, and inflammatory markers for prevalent self-reported cardiovascular disease across NHANES cycles: A repeated cross-sectional validation study
Source: Medicine (Baltimore). 2026 Nov 6;105(45):e51425. doi: 10.1097/MD.0000000000051425 (PMC13747496; doi:10.1097/MD.0000000000051425)
Supplement: Supplementary file 6 [file medi-105-e51425-s007.docx]

**Supplemental Table S7**

Incremental value of renal, metabolic, and inflammatory markers for prevalent self-reported cardiovascular disease across NHANES cycles: a repeated cross-sectional validation study

First author: Haoyang Li; second author: Xiang Li

# Table S7. Predictor missingness by model and period

| **period** | **model** | **weight_variable** | **predictor** | **analysis_sample_n** | **missing_n** | **missing_percent** |
| --- | --- | --- | --- | --- | --- | --- |
| Development 2005-2018 | model0_core | submission_primary_weight | age | 37482 | 0 | 0.0 |
| Development 2005-2018 | model0_core | submission_primary_weight | sex | 37482 | 0 | 0.0 |
| Development 2005-2018 | model0_core | submission_primary_weight | race_ethnicity | 37482 | 0 | 0.0 |
| Development 2005-2018 | model0_core | submission_primary_weight | education | 37482 | 50 | 0.13 |
| Development 2005-2018 | model0_core | submission_primary_weight | smoking | 37482 | 27 | 0.07 |
| Development 2005-2018 | model0_core | submission_primary_weight | bmi | 37482 | 563 | 1.5 |
| Development 2005-2018 | model0_core | submission_primary_weight | mean_sbp | 37482 | 1632 | 4.35 |
| Development 2005-2018 | model0_core | submission_primary_weight | diabetes | 37482 | 0 | 0.0 |
| Development 2005-2018 | model0_core | submission_primary_weight | total_cholesterol | 37482 | 2268 | 6.05 |
| Development 2005-2018 | model0_core | submission_primary_weight | hdl_cholesterol | 37482 | 2268 | 6.05 |
| Development 2005-2018 | model1_renal | submission_primary_weight | age | 37482 | 0 | 0.0 |
| Development 2005-2018 | model1_renal | submission_primary_weight | sex | 37482 | 0 | 0.0 |
| Development 2005-2018 | model1_renal | submission_primary_weight | race_ethnicity | 37482 | 0 | 0.0 |
| Development 2005-2018 | model1_renal | submission_primary_weight | education | 37482 | 50 | 0.13 |
| Development 2005-2018 | model1_renal | submission_primary_weight | smoking | 37482 | 27 | 0.07 |
| Development 2005-2018 | model1_renal | submission_primary_weight | bmi | 37482 | 563 | 1.5 |
| Development 2005-2018 | model1_renal | submission_primary_weight | mean_sbp | 37482 | 1632 | 4.35 |
| Development 2005-2018 | model1_renal | submission_primary_weight | diabetes | 37482 | 0 | 0.0 |
| Development 2005-2018 | model1_renal | submission_primary_weight | total_cholesterol | 37482 | 2268 | 6.05 |
| Development 2005-2018 | model1_renal | submission_primary_weight | hdl_cholesterol | 37482 | 2268 | 6.05 |
| Development 2005-2018 | model1_renal | submission_primary_weight | egfr | 37482 | 2385 | 6.36 |
| Development 2005-2018 | model1_renal | submission_primary_weight | log2_uacr | 37482 | 952 | 2.54 |
| Development 2005-2018 | model2_metabolic | submission_fasting_weight | age | 15956 | 0 | 0.0 |
| Development 2005-2018 | model2_metabolic | submission_fasting_weight | sex | 15956 | 0 | 0.0 |
| Development 2005-2018 | model2_metabolic | submission_fasting_weight | race_ethnicity | 15956 | 0 | 0.0 |
| Development 2005-2018 | model2_metabolic | submission_fasting_weight | education | 15956 | 16 | 0.1 |
| Development 2005-2018 | model2_metabolic | submission_fasting_weight | smoking | 15956 | 15 | 0.09 |
| Development 2005-2018 | model2_metabolic | submission_fasting_weight | bmi | 15956 | 199 | 1.25 |
| Development 2005-2018 | model2_metabolic | submission_fasting_weight | mean_sbp | 15956 | 545 | 3.42 |
| Development 2005-2018 | model2_metabolic | submission_fasting_weight | diabetes | 15956 | 0 | 0.0 |
| Development 2005-2018 | model2_metabolic | submission_fasting_weight | total_cholesterol | 15956 | 152 | 0.95 |
| Development 2005-2018 | model2_metabolic | submission_fasting_weight | hdl_cholesterol | 15956 | 152 | 0.95 |
| Development 2005-2018 | model2_metabolic | submission_fasting_weight | tyg_wc | 15956 | 749 | 4.69 |
| Development 2005-2018 | model3_inflammatory | submission_primary_weight | age | 37482 | 0 | 0.0 |
| Development 2005-2018 | model3_inflammatory | submission_primary_weight | sex | 37482 | 0 | 0.0 |
| Development 2005-2018 | model3_inflammatory | submission_primary_weight | race_ethnicity | 37482 | 0 | 0.0 |
| Development 2005-2018 | model3_inflammatory | submission_primary_weight | education | 37482 | 50 | 0.13 |
| Development 2005-2018 | model3_inflammatory | submission_primary_weight | smoking | 37482 | 27 | 0.07 |
| Development 2005-2018 | model3_inflammatory | submission_primary_weight | bmi | 37482 | 563 | 1.5 |
| Development 2005-2018 | model3_inflammatory | submission_primary_weight | mean_sbp | 37482 | 1632 | 4.35 |
| Development 2005-2018 | model3_inflammatory | submission_primary_weight | diabetes | 37482 | 0 | 0.0 |
| Development 2005-2018 | model3_inflammatory | submission_primary_weight | total_cholesterol | 37482 | 2268 | 6.05 |
| Development 2005-2018 | model3_inflammatory | submission_primary_weight | hdl_cholesterol | 37482 | 2268 | 6.05 |
| Development 2005-2018 | model3_inflammatory | submission_primary_weight | siri | 37482 | 1927 | 5.14 |
| Development 2005-2018 | model4_combined | submission_fasting_weight | age | 15956 | 0 | 0.0 |
| Development 2005-2018 | model4_combined | submission_fasting_weight | sex | 15956 | 0 | 0.0 |
| Development 2005-2018 | model4_combined | submission_fasting_weight | race_ethnicity | 15956 | 0 | 0.0 |
| Development 2005-2018 | model4_combined | submission_fasting_weight | education | 15956 | 16 | 0.1 |
| Development 2005-2018 | model4_combined | submission_fasting_weight | smoking | 15956 | 15 | 0.09 |
| Development 2005-2018 | model4_combined | submission_fasting_weight | bmi | 15956 | 199 | 1.25 |
| Development 2005-2018 | model4_combined | submission_fasting_weight | mean_sbp | 15956 | 545 | 3.42 |
| Development 2005-2018 | model4_combined | submission_fasting_weight | diabetes | 15956 | 0 | 0.0 |
| Development 2005-2018 | model4_combined | submission_fasting_weight | total_cholesterol | 15956 | 152 | 0.95 |
| Development 2005-2018 | model4_combined | submission_fasting_weight | hdl_cholesterol | 15956 | 152 | 0.95 |
| Development 2005-2018 | model4_combined | submission_fasting_weight | egfr | 15956 | 203 | 1.27 |
| Development 2005-2018 | model4_combined | submission_fasting_weight | log2_uacr | 15956 | 193 | 1.21 |
| Development 2005-2018 | model4_combined | submission_fasting_weight | tyg_wc | 15956 | 749 | 4.69 |
| Development 2005-2018 | model4_combined | submission_fasting_weight | siri | 15956 | 64 | 0.4 |
| Temporal 2021-2023 | model0_core | submission_primary_weight | age | 5755 | 0 | 0.0 |
| Temporal 2021-2023 | model0_core | submission_primary_weight | sex | 5755 | 0 | 0.0 |
| Temporal 2021-2023 | model0_core | submission_primary_weight | race_ethnicity | 5755 | 0 | 0.0 |
| Temporal 2021-2023 | model0_core | submission_primary_weight | education | 5755 | 2 | 0.03 |
| Temporal 2021-2023 | model0_core | submission_primary_weight | smoking | 5755 | 7 | 0.12 |
| Temporal 2021-2023 | model0_core | submission_primary_weight | bmi | 5755 | 81 | 1.41 |
| Temporal 2021-2023 | model0_core | submission_primary_weight | mean_sbp | 5755 | 181 | 3.15 |
| Temporal 2021-2023 | model0_core | submission_primary_weight | diabetes | 5755 | 0 | 0.0 |
| Temporal 2021-2023 | model0_core | submission_primary_weight | total_cholesterol | 5755 | 293 | 5.09 |
| Temporal 2021-2023 | model0_core | submission_primary_weight | hdl_cholesterol | 5755 | 293 | 5.09 |
| Temporal 2021-2023 | model1_renal | submission_primary_weight | age | 5755 | 0 | 0.0 |
| Temporal 2021-2023 | model1_renal | submission_primary_weight | sex | 5755 | 0 | 0.0 |
| Temporal 2021-2023 | model1_renal | submission_primary_weight | race_ethnicity | 5755 | 0 | 0.0 |
| Temporal 2021-2023 | model1_renal | submission_primary_weight | education | 5755 | 2 | 0.03 |
| Temporal 2021-2023 | model1_renal | submission_primary_weight | smoking | 5755 | 7 | 0.12 |
| Temporal 2021-2023 | model1_renal | submission_primary_weight | bmi | 5755 | 81 | 1.41 |
| Temporal 2021-2023 | model1_renal | submission_primary_weight | mean_sbp | 5755 | 181 | 3.15 |
| Temporal 2021-2023 | model1_renal | submission_primary_weight | diabetes | 5755 | 0 | 0.0 |
| Temporal 2021-2023 | model1_renal | submission_primary_weight | total_cholesterol | 5755 | 293 | 5.09 |
| Temporal 2021-2023 | model1_renal | submission_primary_weight | hdl_cholesterol | 5755 | 293 | 5.09 |
| Temporal 2021-2023 | model1_renal | submission_primary_weight | egfr | 5755 | 348 | 6.05 |
| Temporal 2021-2023 | model1_renal | submission_primary_weight | log2_uacr | 5755 | 112 | 1.95 |
| Temporal 2021-2023 | model2_metabolic | submission_fasting_weight | age | 2940 | 0 | 0.0 |
| Temporal 2021-2023 | model2_metabolic | submission_fasting_weight | sex | 2940 | 0 | 0.0 |
| Temporal 2021-2023 | model2_metabolic | submission_fasting_weight | race_ethnicity | 2940 | 0 | 0.0 |
| Temporal 2021-2023 | model2_metabolic | submission_fasting_weight | education | 2940 | 0 | 0.0 |
| Temporal 2021-2023 | model2_metabolic | submission_fasting_weight | smoking | 2940 | 3 | 0.1 |
| Temporal 2021-2023 | model2_metabolic | submission_fasting_weight | bmi | 2940 | 33 | 1.12 |
| Temporal 2021-2023 | model2_metabolic | submission_fasting_weight | mean_sbp | 2940 | 93 | 3.16 |
| Temporal 2021-2023 | model2_metabolic | submission_fasting_weight | diabetes | 2940 | 0 | 0.0 |
| Temporal 2021-2023 | model2_metabolic | submission_fasting_weight | total_cholesterol | 2940 | 118 | 4.01 |
| Temporal 2021-2023 | model2_metabolic | submission_fasting_weight | hdl_cholesterol | 2940 | 118 | 4.01 |
| Temporal 2021-2023 | model2_metabolic | submission_fasting_weight | tyg_wc | 2940 | 230 | 7.82 |
| Temporal 2021-2023 | model3_inflammatory | submission_primary_weight | age | 5755 | 0 | 0.0 |
| Temporal 2021-2023 | model3_inflammatory | submission_primary_weight | sex | 5755 | 0 | 0.0 |
| Temporal 2021-2023 | model3_inflammatory | submission_primary_weight | race_ethnicity | 5755 | 0 | 0.0 |
| Temporal 2021-2023 | model3_inflammatory | submission_primary_weight | education | 5755 | 2 | 0.03 |
| Temporal 2021-2023 | model3_inflammatory | submission_primary_weight | smoking | 5755 | 7 | 0.12 |
| Temporal 2021-2023 | model3_inflammatory | submission_primary_weight | bmi | 5755 | 81 | 1.41 |
| Temporal 2021-2023 | model3_inflammatory | submission_primary_weight | mean_sbp | 5755 | 181 | 3.15 |
| Temporal 2021-2023 | model3_inflammatory | submission_primary_weight | diabetes | 5755 | 0 | 0.0 |
| Temporal 2021-2023 | model3_inflammatory | submission_primary_weight | total_cholesterol | 5755 | 293 | 5.09 |
| Temporal 2021-2023 | model3_inflammatory | submission_primary_weight | hdl_cholesterol | 5755 | 293 | 5.09 |
| Temporal 2021-2023 | model3_inflammatory | submission_primary_weight | siri | 5755 | 34 | 0.59 |
| Temporal 2021-2023 | model4_combined | submission_fasting_weight | age | 2940 | 0 | 0.0 |
| Temporal 2021-2023 | model4_combined | submission_fasting_weight | sex | 2940 | 0 | 0.0 |
| Temporal 2021-2023 | model4_combined | submission_fasting_weight | race_ethnicity | 2940 | 0 | 0.0 |
| Temporal 2021-2023 | model4_combined | submission_fasting_weight | education | 2940 | 0 | 0.0 |
| Temporal 2021-2023 | model4_combined | submission_fasting_weight | smoking | 2940 | 3 | 0.1 |
| Temporal 2021-2023 | model4_combined | submission_fasting_weight | bmi | 2940 | 33 | 1.12 |
| Temporal 2021-2023 | model4_combined | submission_fasting_weight | mean_sbp | 2940 | 93 | 3.16 |
| Temporal 2021-2023 | model4_combined | submission_fasting_weight | diabetes | 2940 | 0 | 0.0 |
| Temporal 2021-2023 | model4_combined | submission_fasting_weight | total_cholesterol | 2940 | 118 | 4.01 |
| Temporal 2021-2023 | model4_combined | submission_fasting_weight | hdl_cholesterol | 2940 | 118 | 4.01 |
| Temporal 2021-2023 | model4_combined | submission_fasting_weight | egfr | 2940 | 149 | 5.07 |
| Temporal 2021-2023 | model4_combined | submission_fasting_weight | log2_uacr | 2940 | 56 | 1.9 |
| Temporal 2021-2023 | model4_combined | submission_fasting_weight | tyg_wc | 2940 | 230 | 7.82 |
| Temporal 2021-2023 | model4_combined | submission_fasting_weight | siri | 2940 | 15 | 0.51 |

Abbreviations. AUROC, area under the receiver operating characteristic curve; CI, confidence interval; CVD, cardiovascular disease; DCA, decision-curve analysis; eGFR, estimated glomerular filtration rate; I2, percentage of total variability attributed to between-cycle heterogeneity; MEC, mobile examination center; MI, multiple imputation; n, number; PR-AUC, area under the precision-recall curve; SIRI, systemic inflammation response index; TyG-WC, triglyceride-glucose waist circumference index; UACR, urine albumin-to-creatinine ratio.
