# Supplementary material for: Incremental value of renal, metabolic, and inflammatory markers for prevalent self-reported cardiovascular disease across NHANES cycles: A repeated cross-sectional validation study
Source: Medicine (Baltimore). 2026 Nov 6;105(45):e51425. doi: 10.1097/MD.0000000000051425 (PMC13747496; doi:10.1097/MD.0000000000051425)
Supplement: Supplementary file 7 [file medi-105-e51425-s008.docx]

**Supplemental Table S12**

Incremental value of renal, metabolic, and inflammatory markers for prevalent self-reported cardiovascular disease across NHANES cycles: a repeated cross-sectional validation study

First author: Haoyang Li; second author: Xiang Li

# Table S12. Original and corrected fasting-domain temporal incremental estimates

| **Analysis version** | **Algorithm** | **Comparison** | **n (events)** | **Delta AUROC (95% CI)** | **Delta PR-AUC (95% CI)** | **Brier improvement (95% CI)** | **Log-loss improvement (95% CI)** |
| --- | --- | --- | --- | --- | --- | --- | --- |
| Initial run: unmatched fasting core | Elastic Net | Metabolic | 3397 (434) | -0.0008 (-0.0057 to 0.0035) | -0.0041 (-0.0171 to 0.0106) | -0.0004 (-0.0011 to 0.0001) | -0.0010 (-0.0033 to 0.0012) |
| Initial run: unmatched fasting core | Elastic Net | Combined | 3397 (434) | 0.0056 (-0.0009 to 0.0115) | 0.0186 (-0.0029 to 0.0374) | 0.0009 (-0.0004 to 0.0021) | 0.0035 (-0.0003 to 0.0069) |
| Initial run: unmatched fasting core | XGBoost | Metabolic | 3397 (434) | -0.0043 (-0.0119 to 0.0024) | -0.0117 (-0.0285 to 0.0009) | -0.0009 (-0.0016 to -0.0002) | -0.0029 (-0.0069 to 0.0006) |
| Initial run: unmatched fasting core | XGBoost | Combined | 3397 (434) | 0.0002 (-0.0067 to 0.0065) | 0.0056 (-0.0234 to 0.0258) | 0.0002 (-0.0010 to 0.0014) | 0.0007 (-0.0034 to 0.0042) |
| Comparator corrected; before weight audit | Elastic Net | Metabolic | 3397 (434) | 0.0010 (-0.0023 to 0.0039) | 0.0023 (-0.0073 to 0.0121) | 0.0000 (-0.0004 to 0.0004) | 0.0005 (-0.0011 to 0.0019) |
| Comparator corrected; before weight audit | Elastic Net | Combined | 3397 (434) | 0.0074 (0.0016 to 0.0124) | 0.0250 (0.0040 to 0.0418) | 0.0014 (0.0001 to 0.0025) | 0.0050 (0.0014 to 0.0082) |
| Comparator corrected; before weight audit | XGBoost | Metabolic | 3397 (434) | -0.0010 (-0.0046 to 0.0024) | -0.0068 (-0.0257 to 0.0125) | -0.0005 (-0.0012 to 0.0002) | -0.0011 (-0.0026 to 0.0003) |
| Comparator corrected; before weight audit | XGBoost | Combined | 3397 (434) | 0.0035 (-0.0032 to 0.0104) | 0.0105 (-0.0120 to 0.0352) | 0.0006 (-0.0008 to 0.0020) | 0.0024 (-0.0015 to 0.0064) |
| Final post-audit analysis | Elastic Net | Metabolic | 2940 (355) | 0.0010 (-0.0023 to 0.0039) | 0.0023 (-0.0073 to 0.0121) | 0.0000 (-0.0004 to 0.0004) | 0.0005 (-0.0011 to 0.0019) |
| Final post-audit analysis | Elastic Net | Combined | 2940 (355) | 0.0074 (0.0016 to 0.0124) | 0.0250 (0.0040 to 0.0418) | 0.0014 (0.0001 to 0.0025) | 0.0050 (0.0014 to 0.0082) |
| Final post-audit analysis | XGBoost | Metabolic | 2940 (355) | -0.0010 (-0.0046 to 0.0024) | -0.0068 (-0.0257 to 0.0125) | -0.0005 (-0.0012 to 0.0002) | -0.0011 (-0.0026 to 0.0003) |
| Final post-audit analysis | XGBoost | Combined | 2940 (355) | 0.0035 (-0.0032 to 0.0104) | 0.0105 (-0.0120 to 0.0352) | 0.0006 (-0.0008 to 0.0020) | 0.0024 (-0.0015 to 0.0064) |

Note. The initial temporal run used a mobile-examination-center-trained core for fasting comparisons. The first correction restored the matched fasting-trained core. The final audit then applied WTSAF2YR eligibility and excluded SAS special-missing weights using the greater-than-1e-8 rule. The final eligibility correction changed the nominal sample size but not the weighted estimates because the excluded special-missing records carried negligible numerical weights. All versions are shown for audit transparency and were not used for temporal model selection.

Abbreviations. AUROC, area under the receiver operating characteristic curve; CI, confidence interval; CVD, cardiovascular disease; DCA, decision-curve analysis; eGFR, estimated glomerular filtration rate; I2, percentage of total variability attributed to between-cycle heterogeneity; MEC, mobile examination center; MI, multiple imputation; n, number; PR-AUC, area under the precision-recall curve; SIRI, systemic inflammation response index; TyG-WC, triglyceride-glucose waist circumference index; UACR, urine albumin-to-creatinine ratio.
