# Supplementary material for: Incremental value of renal, metabolic, and inflammatory markers for prevalent self-reported cardiovascular disease across NHANES cycles: A repeated cross-sectional validation study
Source: Medicine (Baltimore). 2026 Nov 6;105(45):e51425. doi: 10.1097/MD.0000000000051425 (PMC13747496; doi:10.1097/MD.0000000000051425)
Supplement: Supplementary file 8 [file medi-105-e51425-s009.docx]

**Supplemental Table S6**

Incremental value of renal, metabolic, and inflammatory markers for prevalent self-reported cardiovascular disease across NHANES cycles: a repeated cross-sectional validation study

First author: Haoyang Li; second author: Xiang Li

# Table S6. Full post-audit corrected temporal performance in the 2021-2023 National Health and Nutrition Examination Survey

| **Algorithm** | **Model** | **Target sample** | **n (events)** | **AUROC (95% CI)** | **PR-AUC (95% CI)** | **Brier score (95% CI)** | **Log loss (95% CI)** | **Calibration intercept (95% CI)** | **Calibration slope (95% CI)** | **O/E ratio (95% CI)** |
| --- | --- | --- | --- | --- | --- | --- | --- | --- | --- | --- |
| Elastic Net | Model 0 core clinical | phlebotomy subsample | 5,755 (739) | 0.853 (0.831 to 0.875) | 0.398 (0.362 to 0.438) | 0.071 (0.065 to 0.077) | 0.239 (0.223 to 0.257) | -0.080 (-0.276 to 0.133) | 0.990 (0.892 to 1.115) | 0.954 (0.890 to 1.017) |
| Elastic Net | Model 1 renal | phlebotomy subsample | 5,755 (739) | 0.857 (0.835 to 0.879) | 0.420 (0.379 to 0.461) | 0.070 (0.064 to 0.076) | 0.236 (0.221 to 0.253) | -0.077 (-0.280 to 0.135) | 0.990 (0.894 to 1.110) | 0.957 (0.891 to 1.020) |
| Elastic Net | Model 0 paired metabolic sample | fasting subsample | 2,940 (355) | 0.845 (0.815 to 0.877) | 0.393 (0.349 to 0.447) | 0.069 (0.062 to 0.075) | 0.237 (0.216 to 0.258) | -0.210 (-0.449 to 0.065) | 0.958 (0.828 to 1.155) | 0.899 (0.797 to 1.003) |
| Elastic Net | Model 2 metabolic | fasting subsample | 2,940 (355) | 0.845 (0.817 to 0.875) | 0.395 (0.351 to 0.451) | 0.069 (0.062 to 0.075) | 0.236 (0.216 to 0.257) | -0.190 (-0.428 to 0.073) | 0.958 (0.832 to 1.139) | 0.912 (0.809 to 1.018) |
| Elastic Net | Model 3 inflammatory | phlebotomy subsample | 5,755 (739) | 0.855 (0.832 to 0.877) | 0.407 (0.369 to 0.447) | 0.070 (0.065 to 0.077) | 0.238 (0.221 to 0.256) | -0.070 (-0.273 to 0.156) | 0.997 (0.895 to 1.129) | 0.955 (0.892 to 1.018) |
| Elastic Net | Model 0 paired combined sample | fasting subsample | 2,940 (355) | 0.845 (0.815 to 0.877) | 0.393 (0.349 to 0.447) | 0.069 (0.062 to 0.075) | 0.237 (0.216 to 0.258) | -0.210 (-0.449 to 0.065) | 0.958 (0.828 to 1.155) | 0.899 (0.797 to 1.003) |
| Elastic Net | Model 4 combined biomarkers | fasting subsample | 2,940 (355) | 0.852 (0.824 to 0.881) | 0.418 (0.370 to 0.472) | 0.068 (0.061 to 0.074) | 0.232 (0.212 to 0.252) | -0.162 (-0.399 to 0.088) | 0.963 (0.841 to 1.127) | 0.927 (0.822 to 1.029) |
| XGBoost | Model 0 core clinical | phlebotomy subsample | 5,755 (739) | 0.862 (0.841 to 0.883) | 0.423 (0.375 to 0.473) | 0.069 (0.064 to 0.076) | 0.234 (0.218 to 0.252) | -0.128 (-0.325 to 0.071) | 0.981 (0.882 to 1.103) | 0.932 (0.865 to 0.998) |
| XGBoost | Model 1 renal | phlebotomy subsample | 5,755 (739) | 0.864 (0.842 to 0.887) | 0.431 (0.380 to 0.482) | 0.069 (0.063 to 0.075) | 0.232 (0.216 to 0.250) | -0.109 (-0.318 to 0.104) | 0.991 (0.889 to 1.118) | 0.935 (0.872 to 0.997) |
| XGBoost | Model 0 paired metabolic sample | fasting subsample | 2,940 (355) | 0.853 (0.823 to 0.887) | 0.432 (0.387 to 0.482) | 0.067 (0.060 to 0.073) | 0.231 (0.208 to 0.253) | -0.153 (-0.374 to 0.095) | 1.015 (0.887 to 1.207) | 0.884 (0.785 to 0.987) |
| XGBoost | Model 2 metabolic | fasting subsample | 2,940 (355) | 0.852 (0.823 to 0.884) | 0.425 (0.368 to 0.485) | 0.067 (0.061 to 0.074) | 0.232 (0.209 to 0.254) | -0.152 (-0.382 to 0.087) | 1.007 (0.877 to 1.199) | 0.891 (0.785 to 0.999) |
| XGBoost | Model 3 inflammatory | phlebotomy subsample | 5,755 (739) | 0.864 (0.842 to 0.886) | 0.431 (0.382 to 0.482) | 0.069 (0.063 to 0.075) | 0.232 (0.216 to 0.250) | -0.076 (-0.281 to 0.140) | 1.018 (0.912 to 1.149) | 0.931 (0.868 to 0.997) |
| XGBoost | Model 0 paired combined sample | fasting subsample | 2,940 (355) | 0.853 (0.823 to 0.887) | 0.432 (0.387 to 0.482) | 0.067 (0.060 to 0.073) | 0.231 (0.208 to 0.253) | -0.153 (-0.374 to 0.095) | 1.015 (0.887 to 1.207) | 0.884 (0.785 to 0.987) |
| XGBoost | Model 4 combined biomarkers | fasting subsample | 2,940 (355) | 0.856 (0.828 to 0.888) | 0.442 (0.395 to 0.493) | 0.066 (0.060 to 0.073) | 0.229 (0.207 to 0.250) | -0.179 (-0.421 to 0.083) | 0.966 (0.846 to 1.137) | 0.914 (0.813 to 1.011) |

Abbreviations. AUROC, area under the receiver operating characteristic curve; CI, confidence interval; CVD, cardiovascular disease; DCA, decision-curve analysis; eGFR, estimated glomerular filtration rate; I2, percentage of total variability attributed to between-cycle heterogeneity; MEC, mobile examination center; MI, multiple imputation; n, number; PR-AUC, area under the precision-recall curve; SIRI, systemic inflammation response index; TyG-WC, triglyceride-glucose waist circumference index; UACR, urine albumin-to-creatinine ratio. O/E, observed-to-expected ratio.
