# Supplementary material for: Incremental value of renal, metabolic, and inflammatory markers for prevalent self-reported cardiovascular disease across NHANES cycles: A repeated cross-sectional validation study
Source: Medicine (Baltimore). 2026 Nov 6;105(45):e51425. doi: 10.1097/MD.0000000000051425 (PMC13747496; doi:10.1097/MD.0000000000051425)
Supplement: Supplementary file 9 [file medi-105-e51425-s010.docx]

**Supplemental Table S1**

Incremental value of renal, metabolic, and inflammatory markers for prevalent self-reported cardiovascular disease across NHANES cycles: a repeated cross-sectional validation study

First author: Haoyang Li; second author: Xiang Li

# Table S1. Sensitivity of renal incremental value to urine albumin-to-creatinine ratio missing-data handling

| **UACR method** | **Algorithm** | **Comparison** | **Delta AUROC, median [range]** | **Delta PR-AUC, median [range]** | **Brier improvement, median [range]** | **Log-loss improvement, median [range]** |
| --- | --- | --- | --- | --- | --- | --- |
| complete case refit | Elastic Net | Combined (Model 4 vs paired Model 0) | 0.0063 [0.0018, 0.0151] | 0.0228 [0.0064, 0.0509] | 0.0014 [0.0003, 0.0027] | 0.0041 [0.0003, 0.0094] |
| main fold median | Elastic Net | Combined (Model 4 vs paired Model 0) | 0.0067 [0.0037, 0.0149] | 0.0220 [0.0034, 0.0502] | 0.0015 [0.0006, 0.0027] | 0.0041 [0.0012, 0.0095] |
| nested multiple imputation | Elastic Net | Combined (Model 4 vs paired Model 0) | 0.0069 [0.0036, 0.0152] | 0.0239 [0.0026, 0.0535] | 0.0016 [0.0006, 0.0029] | 0.0045 [0.0012, 0.0099] |
| stabilized ipw complete case | Elastic Net | Combined (Model 4 vs paired Model 0) | 0.0063 [0.0017, 0.0152] | 0.0239 [0.0057, 0.0514] | 0.0014 [0.0003, 0.0027] | 0.0042 [0.0003, 0.0095] |
| complete case refit | Elastic Net | Renal (Model 1 vs Model 0) | 0.0043 [0.0017, 0.0073] | 0.0186 [0.0021, 0.0266] | 0.0011 [0.0001, 0.0015] | 0.0032 [0.0006, 0.0043] |
| main fold median | Elastic Net | Renal (Model 1 vs Model 0) | 0.0046 [0.0022, 0.0067] | 0.0179 [0.0013, 0.0252] | 0.0011 [0.0002, 0.0015] | 0.0029 [0.0008, 0.0041] |
| nested multiple imputation | Elastic Net | Renal (Model 1 vs Model 0) | 0.0050 [0.0022, 0.0072] | 0.0197 [0.0012, 0.0271] | 0.0012 [0.0002, 0.0017] | 0.0033 [0.0009, 0.0045] |
| stabilized ipw complete case | Elastic Net | Renal (Model 1 vs Model 0) | 0.0047 [0.0018, 0.0076] | 0.0185 [0.0020, 0.0272] | 0.0013 [0.0002, 0.0015] | 0.0035 [0.0007, 0.0044] |
| complete case refit | XGBoost | Combined (Model 4 vs paired Model 0) | 0.0042 [-0.0061, 0.0116] | 0.0262 [-0.0141, 0.0613] | 0.0014 [-0.0013, 0.0027] | 0.0034 [-0.0048, 0.0088] |
| main fold median | XGBoost | Combined (Model 4 vs paired Model 0) | 0.0059 [-0.0081, 0.0110] | 0.0248 [-0.0188, 0.0583] | 0.0014 [-0.0014, 0.0028] | 0.0036 [-0.0057, 0.0086] |
| nested multiple imputation | XGBoost | Combined (Model 4 vs paired Model 0) | 0.0062 [-0.0070, 0.0093] | 0.0263 [-0.0178, 0.0601] | 0.0012 [-0.0011, 0.0028] | 0.0039 [-0.0047, 0.0081] |
| stabilized ipw complete case | XGBoost | Combined (Model 4 vs paired Model 0) | 0.0046 [-0.0055, 0.0115] | 0.0284 [-0.0101, 0.0585] | 0.0015 [-0.0011, 0.0027] | 0.0030 [-0.0042, 0.0088] |
| complete case refit | XGBoost | Renal (Model 1 vs Model 0) | 0.0033 [0.0014, 0.0054] | 0.0129 [0.0008, 0.0259] | 0.0006 [0.0000, 0.0014] | 0.0022 [0.0003, 0.0033] |
| main fold median | XGBoost | Renal (Model 1 vs Model 0) | 0.0025 [0.0017, 0.0050] | 0.0079 [-0.0040, 0.0206] | 0.0007 [0.0001, 0.0016] | 0.0019 [0.0006, 0.0033] |
| nested multiple imputation | XGBoost | Renal (Model 1 vs Model 0) | 0.0024 [0.0014, 0.0049] | 0.0130 [-0.0025, 0.0245] | 0.0008 [0.0001, 0.0016] | 0.0023 [0.0006, 0.0034] |
| stabilized ipw complete case | XGBoost | Renal (Model 1 vs Model 0) | 0.0033 [0.0012, 0.0052] | 0.0128 [-0.0023, 0.0242] | 0.0008 [-0.0000, 0.0015] | 0.0023 [0.0003, 0.0041] |

Abbreviations. AUROC, area under the receiver operating characteristic curve; CI, confidence interval; CVD, cardiovascular disease; DCA, decision-curve analysis; eGFR, estimated glomerular filtration rate; I2, percentage of total variability attributed to between-cycle heterogeneity; MEC, mobile examination center; MI, multiple imputation; n, number; PR-AUC, area under the precision-recall curve; SIRI, systemic inflammation response index; TyG-WC, triglyceride-glucose waist circumference index; UACR, urine albumin-to-creatinine ratio.
