# Supplementary material for: Incremental value of renal, metabolic, and inflammatory markers for prevalent self-reported cardiovascular disease across NHANES cycles: A repeated cross-sectional validation study
Source: Medicine (Baltimore). 2026 Nov 6;105(45):e51425. doi: 10.1097/MD.0000000000051425 (PMC13747496; doi:10.1097/MD.0000000000051425)
Supplement: Supplementary file 10 [file medi-105-e51425-s012.docx]

**Supplemental Table S2**

Incremental value of renal, metabolic, and inflammatory markers for prevalent self-reported cardiovascular disease across NHANES cycles: a repeated cross-sectional validation study

First author: Haoyang Li; second author: Xiang Li

# Table S2. Sensitivity of incremental value to class-imbalance weighting

| **Imbalance setting** | **Algorithm** | **Comparison** | **Delta AUROC, median [range]** | **Delta PR-AUC, median [range]** | **Brier improvement, median [range]** | **Log-loss improvement, median [range]** |
| --- | --- | --- | --- | --- | --- | --- |
| class weighted refit | Elastic Net | Combined (Model 4 vs paired Model 0) | 0.0065 [0.0037, 0.0150] | 0.0244 [0.0009, 0.0466] | 0.0067 [0.0011, 0.0073] | 0.0139 [0.0006, 0.0146] |
| main observed class distribution | Elastic Net | Combined (Model 4 vs paired Model 0) | 0.0067 [0.0037, 0.0149] | 0.0220 [0.0034, 0.0502] | 0.0015 [0.0006, 0.0027] | 0.0041 [0.0012, 0.0095] |
| class weighted refit | Elastic Net | Inflammatory (Model 3 vs Model 0) | 0.0009 [-0.0000, 0.0022] | 0.0040 [-0.0061, 0.0095] | 0.0012 [-0.0007, 0.0017] | 0.0023 [-0.0031, 0.0037] |
| main observed class distribution | Elastic Net | Inflammatory (Model 3 vs Model 0) | 0.0009 [0.0002, 0.0030] | 0.0036 [-0.0048, 0.0075] | 0.0002 [-0.0002, 0.0003] | 0.0006 [-0.0002, 0.0014] |
| class weighted refit | Elastic Net | Metabolic (Model 2 vs paired Model 0) | 0.0008 [-0.0004, 0.0037] | -0.0004 [-0.0040, 0.0039] | 0.0001 [-0.0004, 0.0013] | 0.0009 [-0.0007, 0.0037] |
| main observed class distribution | Elastic Net | Metabolic (Model 2 vs paired Model 0) | 0.0013 [-0.0001, 0.0034] | 0.0006 [-0.0043, 0.0050] | 0.0002 [-0.0003, 0.0004] | 0.0003 [-0.0007, 0.0015] |
| class weighted refit | Elastic Net | Renal (Model 1 vs Model 0) | 0.0043 [0.0020, 0.0070] | 0.0194 [0.0024, 0.0254] | 0.0038 [0.0006, 0.0050] | 0.0073 [-0.0011, 0.0097] |
| main observed class distribution | Elastic Net | Renal (Model 1 vs Model 0) | 0.0046 [0.0022, 0.0067] | 0.0179 [0.0013, 0.0252] | 0.0011 [0.0002, 0.0015] | 0.0029 [0.0008, 0.0041] |
| class weighted refit | XGBoost | Combined (Model 4 vs paired Model 0) | 0.0029 [-0.0044, 0.0116] | 0.0328 [-0.0135, 0.0483] | 0.0048 [0.0020, 0.0067] | 0.0115 [0.0041, 0.0182] |
| main observed class distribution | XGBoost | Combined (Model 4 vs paired Model 0) | 0.0059 [-0.0081, 0.0110] | 0.0248 [-0.0188, 0.0583] | 0.0014 [-0.0014, 0.0028] | 0.0036 [-0.0057, 0.0086] |
| class weighted refit | XGBoost | Inflammatory (Model 3 vs Model 0) | 0.0006 [-0.0009, 0.0026] | -0.0003 [-0.0058, 0.0061] | 0.0010 [-0.0019, 0.0034] | 0.0018 [-0.0097, 0.0084] |
| main observed class distribution | XGBoost | Inflammatory (Model 3 vs Model 0) | 0.0007 [-0.0008, 0.0020] | 0.0011 [-0.0062, 0.0107] | 0.0002 [-0.0007, 0.0004] | 0.0004 [-0.0008, 0.0008] |
| class weighted refit | XGBoost | Metabolic (Model 2 vs paired Model 0) | 0.0012 [-0.0032, 0.0042] | 0.0047 [-0.0109, 0.0073] | 0.0006 [-0.0008, 0.0009] | 0.0007 [-0.0027, 0.0064] |
| main observed class distribution | XGBoost | Metabolic (Model 2 vs paired Model 0) | -0.0006 [-0.0024, 0.0045] | -0.0060 [-0.0125, 0.0121] | -0.0003 [-0.0017, 0.0004] | -0.0004 [-0.0039, 0.0023] |
| class weighted refit | XGBoost | Renal (Model 1 vs Model 0) | 0.0027 [0.0014, 0.0070] | 0.0112 [-0.0035, 0.0219] | 0.0034 [0.0018, 0.0047] | 0.0069 [0.0006, 0.0102] |
| main observed class distribution | XGBoost | Renal (Model 1 vs Model 0) | 0.0025 [0.0017, 0.0050] | 0.0079 [-0.0040, 0.0206] | 0.0007 [0.0001, 0.0016] | 0.0019 [0.0006, 0.0033] |

Abbreviations. AUROC, area under the receiver operating characteristic curve; CI, confidence interval; CVD, cardiovascular disease; DCA, decision-curve analysis; eGFR, estimated glomerular filtration rate; I2, percentage of total variability attributed to between-cycle heterogeneity; MEC, mobile examination center; MI, multiple imputation; n, number; PR-AUC, area under the precision-recall curve; SIRI, systemic inflammation response index; TyG-WC, triglyceride-glucose waist circumference index; UACR, urine albumin-to-creatinine ratio.
