# Supplementary material for: Incremental value of renal, metabolic, and inflammatory markers for prevalent self-reported cardiovascular disease across NHANES cycles: A repeated cross-sectional validation study
Source: Medicine (Baltimore). 2026 Nov 6;105(45):e51425. doi: 10.1097/MD.0000000000051425 (PMC13747496; doi:10.1097/MD.0000000000051425)
Supplement: Supplementary file 11 [file medi-105-e51425-s013.docx]

**Supplemental Table S8**

Incremental value of renal, metabolic, and inflammatory markers for prevalent self-reported cardiovascular disease across NHANES cycles: a repeated cross-sectional validation study

First author: Haoyang Li; second author: Xiang Li

# Table S8. Strict complete-negative cardiovascular disease outcome sensitivity

| **Period** | **Strict n (events)** | **Delta AUROC (95% CI)** | **Delta PR-AUC (95% CI)** | **Brier improvement (95% CI)** | **Log-loss improvement (95% CI)** |
| --- | --- | --- | --- | --- | --- |
| development 2005-2018 out-of-cycle | 37308 (4222) | 0.0046 (0.0030 to 0.0062) | 0.0165 (0.0104 to 0.0228) | 0.0009 (0.0006 to 0.0012) | 0.0028 (0.0019 to 0.0037) |
| temporal 2021-2023 | 5728 (739) | 0.0041 (-0.0001 to 0.0081) | 0.0216 (0.0090 to 0.0320) | 0.0010 (0.0002 to 0.0018) | 0.0030 (0.0005 to 0.0055) |

Abbreviations. AUROC, area under the receiver operating characteristic curve; CI, confidence interval; CVD, cardiovascular disease; DCA, decision-curve analysis; eGFR, estimated glomerular filtration rate; I2, percentage of total variability attributed to between-cycle heterogeneity; MEC, mobile examination center; MI, multiple imputation; n, number; PR-AUC, area under the precision-recall curve; SIRI, systemic inflammation response index; TyG-WC, triglyceride-glucose waist circumference index; UACR, urine albumin-to-creatinine ratio.
