# Supplementary material for: Incremental value of renal, metabolic, and inflammatory markers for prevalent self-reported cardiovascular disease across NHANES cycles: A repeated cross-sectional validation study
Source: Medicine (Baltimore). 2026 Nov 6;105(45):e51425. doi: 10.1097/MD.0000000000051425 (PMC13747496; doi:10.1097/MD.0000000000051425)
Supplement: Supplementary file 12 [file medi-105-e51425-s014.docx]

**Supplemental Table S3**

Incremental value of renal, metabolic, and inflammatory markers for prevalent self-reported cardiovascular disease across NHANES cycles: a repeated cross-sectional validation study

First author: Haoyang Li; second author: Xiang Li

# Table S3. Exploratory decision-curve analysis summary

| **Algorithm** | **Comparison** | **Thresholds with extended NB > core** | **Thresholds where extended was best strategy** | **Maximum NB difference (threshold)** | **CI-positive thresholds, n/30** | **CI-positive threshold list** |
| --- | --- | --- | --- | --- | --- | --- |
| Elastic Net | Combined (Model 4 vs paired Model 0) | 26/30 | 26/30 | 0.0043 (0.30) | 12/30 | 0.04, 0.14, 0.15, 0.22, 0.23, 0.24, 0.25, 0.26, 0.27, 0.28, 0.29, 0.30 |
| Elastic Net | Inflammatory (Model 3 vs Model 0) | 19/30 | 19/30 | 0.0006 (0.14) | 2/30 | 0.04, 0.10 |
| Elastic Net | Metabolic (Model 2 vs paired Model 0) | 17/30 | 17/30 | 0.0009 (0.23) | 1/30 | 0.04 |
| Elastic Net | Renal (Model 1 vs Model 0) | 29/30 | 29/30 | 0.0021 (0.29) | 16/30 | 0.07, 0.08, 0.13, 0.14, 0.15, 0.16, 0.17, 0.18, 0.19, 0.20, 0.22, 0.23, 0.27, 0.28, 0.29, 0.30 |
| XGBoost | Combined (Model 4 vs paired Model 0) | 25/30 | 25/30 | 0.0031 (0.25) | 9/30 | 0.22, 0.23, 0.24, 0.25, 0.26, 0.27, 0.28, 0.29, 0.30 |
| XGBoost | Inflammatory (Model 3 vs Model 0) | 18/30 | 18/30 | 0.0010 (0.12) | 3/30 | 0.06, 0.11, 0.12 |
| XGBoost | Metabolic (Model 2 vs paired Model 0) | 15/30 | 15/30 | 0.0011 (0.21) | 1/30 | 0.02 |
| XGBoost | Renal (Model 1 vs Model 0) | 27/30 | 27/30 | 0.0018 (0.20) | 11/30 | 0.06, 0.10, 0.12, 0.20, 0.21, 0.24, 0.25, 0.26, 0.27, 0.28, 0.30 |

Abbreviations. AUROC, area under the receiver operating characteristic curve; CI, confidence interval; CVD, cardiovascular disease; DCA, decision-curve analysis; eGFR, estimated glomerular filtration rate; I2, percentage of total variability attributed to between-cycle heterogeneity; MEC, mobile examination center; MI, multiple imputation; n, number; PR-AUC, area under the precision-recall curve; SIRI, systemic inflammation response index; TyG-WC, triglyceride-glucose waist circumference index; UACR, urine albumin-to-creatinine ratio. NB, net benefit.
